# Ion‐Confinement‐Assisted Erasure Purifies Oxidized MXene for Reuse

**DOI:** 10.1002/advs.202522266

**Published:** 2026-01-25

**Authors:** Xuefeng Zhang, Yujia Wu, Huayu Xu, Jinyi Ye, Yanfang Yang, Haohan Wu, Jiayi Lu, Hao Li, Dongxiao Kan, Jingfeng Wang, Hui Kang, Zhimin Fan

**Affiliations:** ^1^ School of Chemistry and Materials Engineering Guangdong Provincial Key Laboratory for Electronic Functional Materials and Devices Huizhou University Huizhou China; ^2^ State Key Laboratory of Advanced Inorganic Fibers and Composites School of Chemistry and Chemical Engineering Harbin Institute of Technology Harbin China; ^3^ Advanced Materials Research Central Northwest Institute for Nonferrous Metal Research Xi'an China; ^4^ Department of Applied Biology and Chemical Technology The Hong Kong Polytechnic University Hong Kong China

**Keywords:** high volumetric capacitance, ion‐confinement effect, oxide erasure‐purification, regenerative recycling, Ti_3_C_2_T_x_ MXene

## Abstract

Titanium carbide (Ti_3_C_2_T*_x_*) MXene has attracted broad interest owing to its versatile functional properties. However, its intrinsic instability in aqueous environments drives spontaneous surface oxidation to insulating TiO_2_, resulting in pronounced performance degradation and posing a major obstacle to practical utilization. Here, we propose an erasure‐purification strategy that selectively targets surface‐derived oxide phases on oxidized MXene. By constructing an ion‐confined interfacial environment followed by a brief acid‐washing step, further oxidation is mitigated while pre‐existing TiO_2_ is selectively removed. The recovered MXene exhibits restored colloidal stability and can be reassembled into conductive films delivering high volumetric capacitance and favorable rate performance. In addition, the purified MXene demonstrates competitive low‐frequency electromagnetic attenuation and low infrared emissivity in assembled forms. This approach is applicable to MXene with different morphologies and oxidation states, offering a practical and generalizable route for reclaiming and reusing oxidized MXene materials.

## Graphical abstract

An erasure‐purification strategy removes surface‐derived TiO_2_ from oxidized Ti_3_C_2_T*_x_* MXene by ion‐confinement‐induced interfacial conversion to soluble titanates, followed by brief acid washing. The scalable process enables approximately 70% material recovery, restores electrical conductivity, and recovers functional performance, including high volumetric capacitance (1801 F cm^−3^), low‐frequency electromagnetic absorption, and competitive infrared stealth.

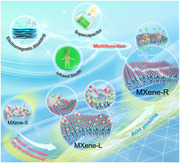

## 1 Introduction

Since its first report in 2011, titanium carbide MXene (Ti_3_C_2_T*_x_*, where T*_x_* denotes surface terminations such as ─OH, ─O, and ─F) has attracted substantial interest owing to its intrinsic hydrophilicity, high electrical conductivity, and favorable mechanical properties [1, 2, 3]. These attributes have enabled a wide range of applications, including electrochemical energy storage, electromagnetic interference shielding, communications, and flexible electronics [4, 5, 6, 7, 8]. Notably, MXene can be produced in a scalable manner through wet chemical etching routes, positioning it among the most promising 2D materials for practical applications [9, 10, 11]. However, freshly synthesized MXene is typically obtained as a fully delaminated monolayer nanosheets dispersed in aqueous media, in which the exposed terminal Ti atoms are highly vulnerable to attack by water and dissolved oxygen, leading to progressive oxidation into electrically insulating TiO_2_. This surface degradation markedly deteriorates the intrinsic properties of MXene. Once partial oxidation occurs, the material can no longer be regarded as MXene in a strict sense and is generally discarded [12]. This issue becomes particularly acute in emerging applications that rely on large‐scale aqueous processing, where unavoidable oxidation constitutes a major impediment to the translation of MXene from laboratory studies to industrial and commercial implementation.

At present, strategies aimed at improving the oxidative stability of aqueous MXene dispersions can be broadly classified into intrinsic regulation approaches and extrinsic protection methods. Intrinsic strategies primarily focus on enhancing crystallographic integrity or tailoring surface terminations to reduce edge defects and improve the reductive stability of transition metal species, thereby increasing oxidation resistance through structural optimization of the MXene itself [13, 14]. In contrast, extrinsic strategies mainly rely on controlling the storage environment to limit the exposure of MXene to oxygen and other oxidative species, thus retarding oxidation during handling and storage [15, 16, 17]. However, theoretical calculations have revealed that the oxidation of MXene in aqueous environments is thermodynamically spontaneous and energetically favorable, as evidenced by a continuous decrease in the system free energy [18, 19]. Consequently, these approaches can at best delay, rather than fundamentally suppress, the oxidative degradation of MXene. Moreover, the TiO_2_ species formed on the surfaces of oxidized MXene exhibit high chemical stability and are extremely difficult to remove using conventional acid, base, or salt treatments [20]. To date, only a limited number of studies have reported the removal of surface‐derived TiO_2_ from degraded MXene through etching with highly toxic hydrofluoric acid [21]; however, such treatments involve serious safety and environmental concerns and are incompatible with the fluorine‐free development trend of MXene materials. In addition, the large‐scale production of MXene is generally accompanied by high energy consumption and considerable environmental burdens [22], which substantially increase manufacturing costs and further hinder commercialization. From the perspectives of cost reduction, circular manufacturing, and sustainable development, the development of efficient purification and regeneration strategies for oxidized MXene has therefore become an urgent and compelling need.

In this work, we develop an erasure–purification strategy to selectively remove surface‐derived oxide species from oxidized Ti_3_C_2_T*_x_* MXene. The structural evolution and assembly behavior of MXene with different oxidation states and morphologies during the erasure process are systematically investigated. On this basis, the changes in colloidal stability, assembly characteristics, and functional performance following oxide removal are further examined, thereby elucidating the scope and limitations of this strategy for the recovery of oxidized MXene. This work provides mechanistic insights into the structure–property evolution of oxidized MXene during recovery and establishes a feasible pathway for the reutilization and circular use of 2D materials.

## 2 Results and Discussion

### 2.1 Microstructural Characteristics of the Erasure‐Purified Oxidized MXene

**FIGURE 1:**
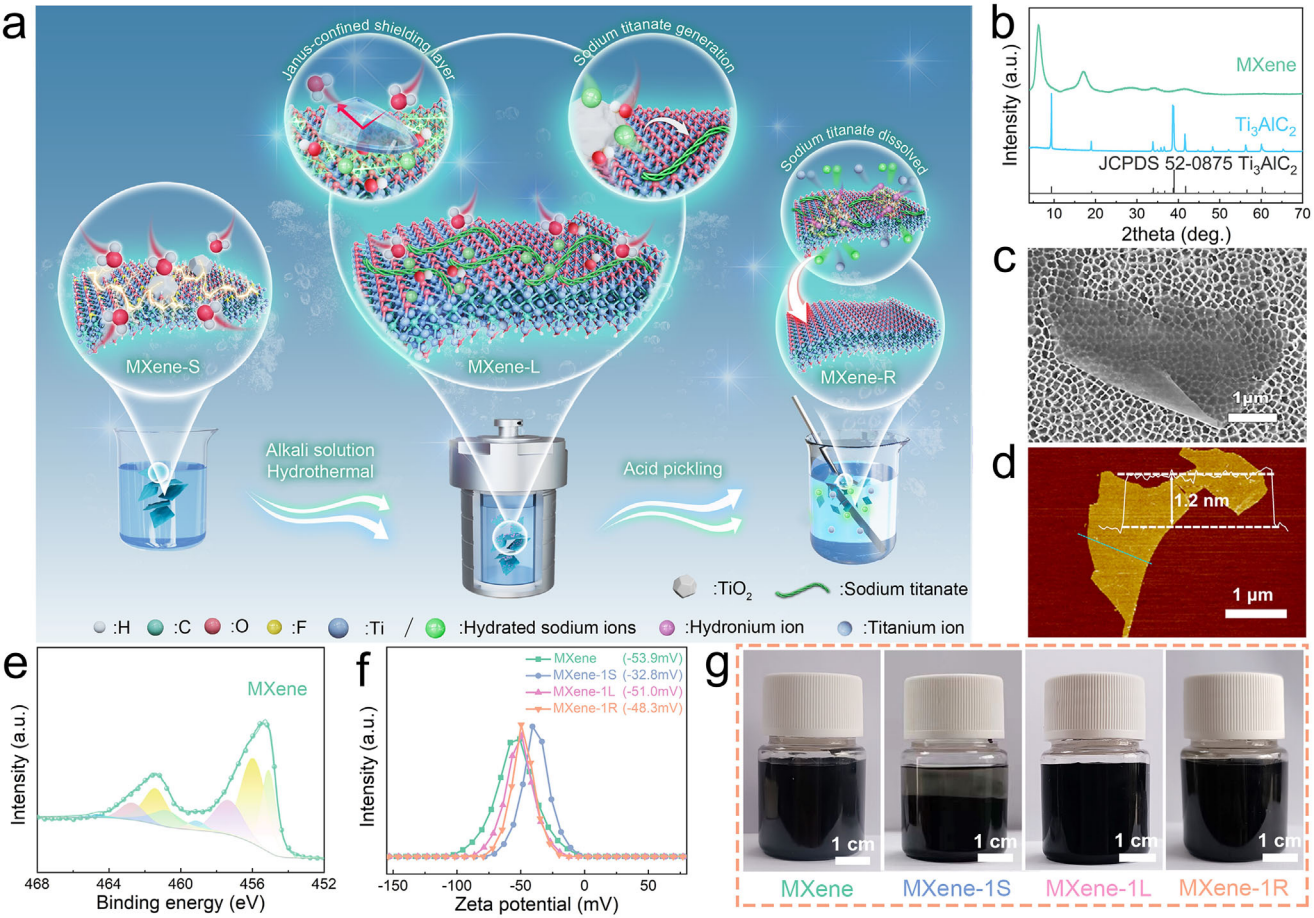
Erasure–purification strategy for failed MXene and recovery of its structural and colloidal characteristics. (a) Schematic illustration of the erasure–purification process and the proposed mechanism for oxidized MXene. (b) XRD patterns of Ti_3_AlC_2_ MAX phase and Ti_3_C_2_T*_x_* MXene. (c) SEM image and (d) AFM image of Ti_3_C_2_T*_x_* nanosheets. (e) High‐resolution Ti 2p binding energy spectra of MXene. (f) Zeta potentials of the samples. (g) Optical images of fresh MXene dispersion and oxidized MXene before and after erasure–purification (2 mg mL^−^^1^ for all samples).

Figure 1a schematically illustrates the overall erasure–purification process designed for the selective removal of surface‐derived oxide species from oxidized Ti_3_C_2_T*_x_* MXene. Through an alkaline hydrothermal treatment followed by a brief acid‐washing step, surface oxide phases formed during oxidation are transformed and removed, providing a structural basis for subsequent recovery and performance restoration. High‐quality monolayer Ti_3_C_2_T*_x_* MXene dispersions were prepared using a modified minimally intensive layer delamination method (Figure S1a,b). Upon dilution, the dispersion exhibited a characteristic dark‐green appearance and a pronounced Tyndall effect (Figure S1c) [23]. X‐ray diffraction (XRD) analysis confirmed that, after etching and delamination, the (104) reflection of the Ti_3_AlC_2_ MAX phase at 39° disappeared completely, while the (002) peak became broadened and shifted toward lower angles, indicating successful exfoliation (Figure 1b) [24]. Microscopic characterization further revealed that the initially dense, layered ceramic precursor (Figure S2) was converted into monolayer nanosheets with an average thickness of approximately 1.2 nm (Figure 1c,d, and Figure S3). The films assembled via vacuum filtration displayed the typical light‐purple coloration of Ti_3_C_2_T*_x_* MXene (Figure S4a), and both the surface and cross‐sectional morphologies (Figure S4b,c) were consistent with those of high‐purity MXene films. Elemental valence analysis (Figure 1e) and Raman spectra (Figure S5) further verified the successful synthesis of high‐quality monolayer MXene, in good agreement with previous reports [25]. To simulate oxidative degradation, the MXene dispersion was diluted to 2 mg mL^−^^1^ and stored at room temperature for 1 and 2 months, yielding samples denoted as MXene‐1S and MXene‐2S, respectively. These oxidized dispersions were subsequently subjected to the erasure–purification treatment, consisting of alkaline hydrothermal processing (suffix “L”) followed by acid washing (suffix “R”). During storage, Ti atoms located at the edges and defect sites of the MXene nanosheets were preferentially attacked by water molecules, leading to the nucleation of TiO_2_. Concurrently, driven by internal electric fields, Ti and C atoms underwent diffusion and local restructuring within defect regions [18, 26], resulting in a gradual decrease in the absolute surface potential (Figure 1f) and promoting nanosheet aggregation through hydrogen bonding and van der Waals interactions (Figure 1g). After two months of storage, these oxidative effects became more pronounced, as evidenced by a substantial reduction in the absolute zeta potential and visible sediment shrinkage accompanied by the formation of a white oxidized layer at the top of the dispersion (Figure S6). In contrast, the erasure‐treated dispersions, MXene‐1R and MXene‐2R, exhibited restored zeta potentials of −48.3 and −49.0 mV, respectively, values close to that of freshly prepared MXene. This recovery of electrostatic repulsion endowed the dispersions with excellent redispersibility, with the colloidal suspension remaining stable and free of sedimentation after one week of static aging, indicating that the erasure–purification strategy effectively regenerated oxidized MXene into a stable and purified colloidal state.

**FIGURE 2:**
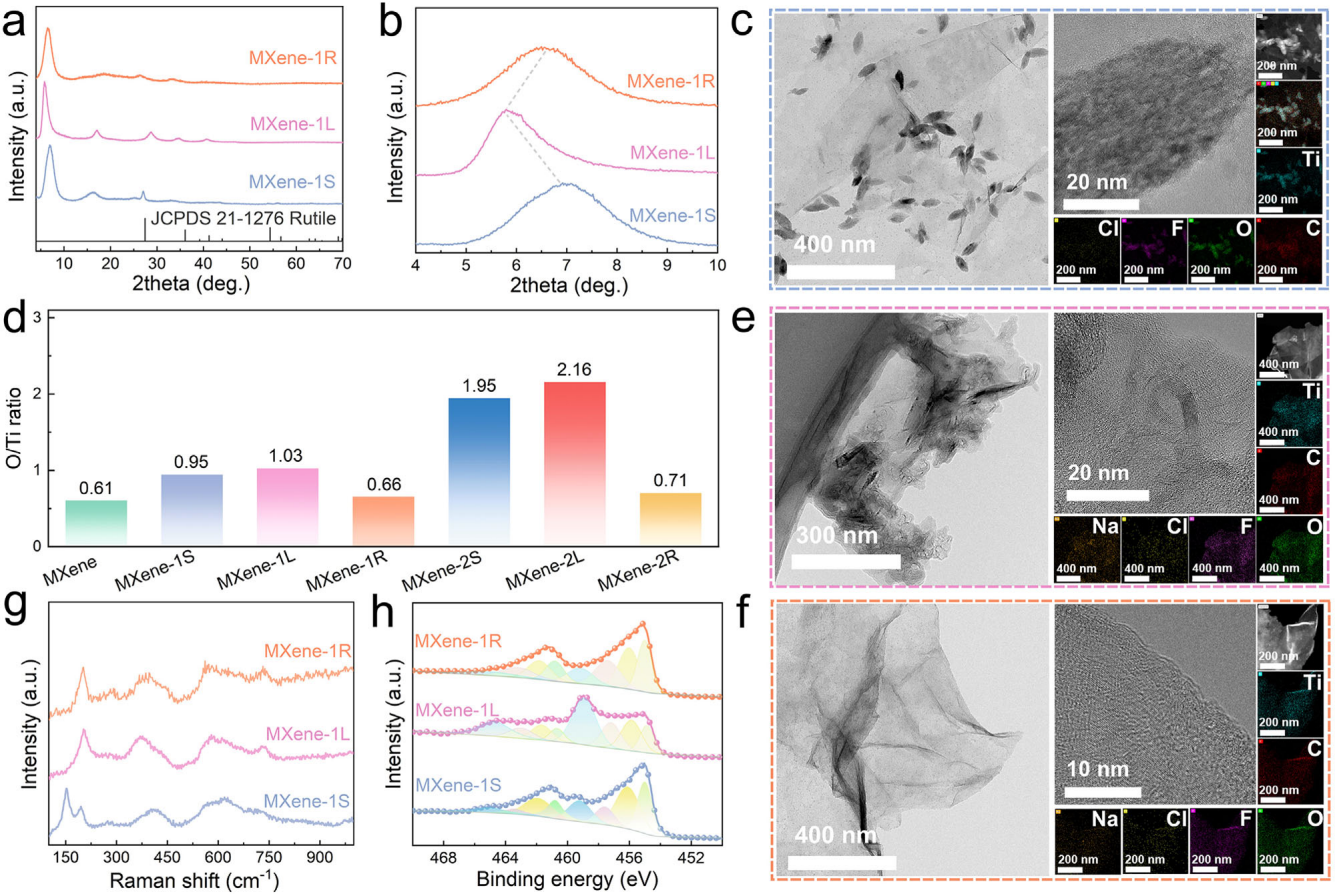
Microstructural evolution during the erasure–purification of oxidized MXene. XRD patterns of (a) MXene‐1S before and after erasure and (b) the corresponding enlarged view of the (002) diffraction peak. (c) TEM image and elemental mappings of MXene‐1S. (d) Atomic O/Ti ratios of fresh MXene and samples before and after erasure. (e) TEM image and elemental mappings of MXene‐1L. (f) TEM image and elemental mappings of MXene‐1R. (g) Raman spectra and (h) high‐resolution Ti 2p binding energy spectra of MXene‐1S, MXene‐1L, and MXene‐1R.

Figure 2a,b present the XRD patterns of MXene stored for 1 month before and after erasure treatment, respectively. For MXene‐1S, a new diffraction peak emerged at approximately 27°, indicative of the gradual formation of rutile‐phase TiO_2_, which is a thermodynamically stable oxidation product [17]. TEM further revealed the presence of sparsely distributed, olive‐like TiO_2_ nanoparticles with an average radial size of approximately 64 nm (Figure 2c; Figure S7a). This oxidation process was accompanied by pronounced enrichment of Ti and O elements, leading to an increase in the O/Ti atomic ratio from 0.61 for fresh MXene to 0.95 (Figure 2d). After 2 months of storage, MXene‐2S was nearly fully converted into rutile TiO_2_ with a larger particle size (Figure S7b). Correspondingly, only a weak residual (002) reflection associated with MXene was observed in the XRD pattern (Figure S8), together with minimal remaining nanosheet features. The O/Ti atomic ratio approached the stoichiometric value of TiO_2_ (Figure 2d), further confirming extensive oxidation. Upon hydrothermal treatment in a saturated alkaline solution, the surface TiO_2_ nanoparticles (Figure 2c and Figure S9a) underwent a distinct morphological transformation into diffuse fibrous nanowires (Figure 2e and Figure S9b). This behavior can be attributed to the highly stable crystal structure of rutile TiO_2_, in which TiO_6_ octahedra are connected through shared edges, rendering the phase resistant to dissolution or rolling into tubular morphologies commonly observed for anatase TiO_2_. Under hydrothermal conditions, however, hydroxide ions disrupted the rutile lattice, inducing its reconstruction into low‐crystallinity fibrous titanate structures. This transformation is corroborated by the near disappearance of diffraction features in the corresponding XRD patterns (Figure 2a and Figure S8) [27, 28]. The expansion of the (002) interlayer spacing from 1.42 to 1.98 nm (Figure 2b) suggests the intercalation of the newly formed sodium titanate nanowires [29]. Energy‐dispersive spectroscopy (EDS) confirmed the incorporation of Na (Figure 2e and Figure S9b), with Na contents of 4.8% and 9.1% for MXene‐1L and MXene‐2L, respectively (Figure S10), accompanied by O/Ti ratios of 1.03 and 2.16 (Figure 2d), only slightly higher than those prior to hydrothermal treatment. These results indicate that the hydrothermal process predominantly involves the conversion of surface TiO_2_ into titanate species, rather than further oxidation of the MXene core. Following acid washing, the fibrous sodium titanate structures were effectively removed, and the MXene recovered a clean and well‐defined 2D morphology. It should be noted that the oxidation and etching processes inevitably introduced a certain degree of in‐plane defects into the erasure‐purified MXene nanosheets (Figure S3, Figure 2f, and Figure S11). Meanwhile, the (002) reflection shifted back toward higher angles (Figure 2b), and Na content decreased to below 2% (Figure S10). This residual Na is likely associated with minor interlayer trapping or experimental uncertainty. The O/Ti ratios of MXene‐1R and MXene‐2R recovered to 0.66 and 0.71, respectively (Figure 2d). Combined with elemental analysis of F and Cl as well as FTIR spectra, the slight increase in O content can be attributable not to residual oxidation but to the substitution of ─F and ─Cl surface terminations (Ti─F and Ti─Cl bond energies of 569 and 213 kJ mol^−^^1^, respectively) by more thermodynamically stable Ti─O species (Ti─O bond energy: 666.5 kJ mol^−^^1^; Figure S12) during hydrothermal treatment [14, 30, 31, 32]. The introduction of these oxygen‐containing terminations is expected to reduce ion diffusion resistance and provide additional pseudocapacitive contributions, thereby establishing a favorable basis for enhanced electrochemical performance [33].

UV–vis absorption spectroscopy is widely employed as a convenient probe to assess the oxidation state of MXenes. Fresh MXene exhibits a characteristic absorption peak at approximately 785 nm, the intensity of which is commonly used to track changes in the oxidation degree [15, 17]. As oxidation progressed, the absorption intensity at 785 nm decreased markedly for MXene‐1S (Figure S13a) and MXene‐2S (Figure S13b). After hydrothermal treatment, MXene‐1L and MXene‐2L still displayed relatively low absorption intensities. In contrast, following the complete erasure–purification treatment and subsequent redispersion, MXene‐1R and MXene‐2R recovered absorption intensities close to that of fresh MXene at the same concentration. This result indicates that the oxidation–recovery process of MXene is, to a certain extent, reversible with respect to its optical absorption characteristics. It is noteworthy that, compared with fresh MXene, the characteristic absorption peak of the erasure–purified MXene exhibited a slight red shift from approximately 785 to around 840 nm. This shift is likely associated with the redistribution of surface terminations or changes in the local chemical environment induced during the hydrothermal process, which may modify the local electronic structure and optical response of MXene. The effective removal of surface oxides and restoration of chemical purity in oxidized MXene were further corroborated by Raman spectroscopy and X‐ray photoelectron spectroscopy (XPS). As shown in Figure 2g and Figure S14a, both MXene‐1S and MXene‐2S exhibited characteristic Raman bands of TiO_2_ near 150 cm^−^^1^, confirming the presence of oxidized phases. For MXene‐1L obtained after hydrothermal treatment, no distinct Raman features corresponding to amorphous sodium titanate were observed, likely due to the weak vibrational signals being masked by the dominant intrinsic Raman response of MXene. In contrast, the more severely oxidized MXene‐2L displayed clear vibrational bands at 276 and 655 cm^−^^1^, which can be assigned to Na─O─Ti lattice vibrations and Ti─O─Ti stretching modes, respectively [27]. These features disappeared completely after HCl acid washing, and the Raman spectra of the resulting MXene‐1R and MXene‐2R closely resembled that of fresh MXene. Notably, the Raman signals associated with Ti─O and Ti─OH surface groups at approximately 289 and 369 cm^−^^1^ were significantly enhanced after the erasure treatment, further indicating the enrichment of oxygen‐containing terminations on the MXene surface [34]. Meanwhile, the increased full width at half maximum of the characteristic peak near 200 cm^−^^1^ suggests an increase in the in‐plane defect density of MXene nanosheets during the erasure–purification process. XPS analysis (Figure 1e, Figure 2h, and Figure S14b) showed a pronounced increase in the Ti^4^^+^ component in oxidized MXene‐1S and MXene‐2S, consistent with the formation of TiO_2_. After erasure treatment, the intensity of these high‐valence components was substantially reduced, approaching the spectral profile of pristine MXene. These spectroscopic results collectively confirm that the proposed erasure strategy enables the transformation and effective removal of TiO_2_ species from oxidized MXene surfaces. Figure S15 displays the surface and cross‐sectional morphologies of films assembled from oxidized and regenerated MXene. Although films derived from MXene‐1S and MXene‐1L retained a stacked lamellar structure, the intercalation of TiO_2_ particles and sodium titanate nanowires led to a significant decrease in film density, from 3.9 g cm^−^^3^ for fresh MXene to 3.0 and 2.2 g cm^−^^3^, respectively (Figure S16). Interestingly, the MXene‐1R film recovered through the erasure process regained a compact lamellar architecture with a density of 3.8 g cm^−^^3^, together with good mechanical flexibility (Figure S17). For the more severely oxidized samples, MXene‐2S and MXene‐2L could not be assembled into continuous films, instead forming disordered aggregates composed of nanosheets and oxidized debris (Figure S18). Nevertheless, after complete erasure treatment, MXene‐2R was successfully reassembled into a densely packed film with a density of 3.4 g cm^−^^3^ (Figure S16). The slightly lower density compared with pristine MXene can be attributed to in‐plane voids created by the removal of TiO_2_ particles, as well as partial nanosheet fragmentation during ultrasonic redispersion (Figure S19). Overall, these results demonstrate that the erasure–purification strategy effectively removes surface oxides from degraded MXene. Although a certain degree of in‐plane defects is unavoidably introduced during the recovery process, the regenerated MXene retains the characteristic crystalline framework and chemical purity close to that of the pristine state, thereby providing a robust basis for subsequent processing, performance recovery, and further practical utilization.

### 2.2 Mechanism of Erasure and Purification of Degraded MXene

**FIGURE 3:**
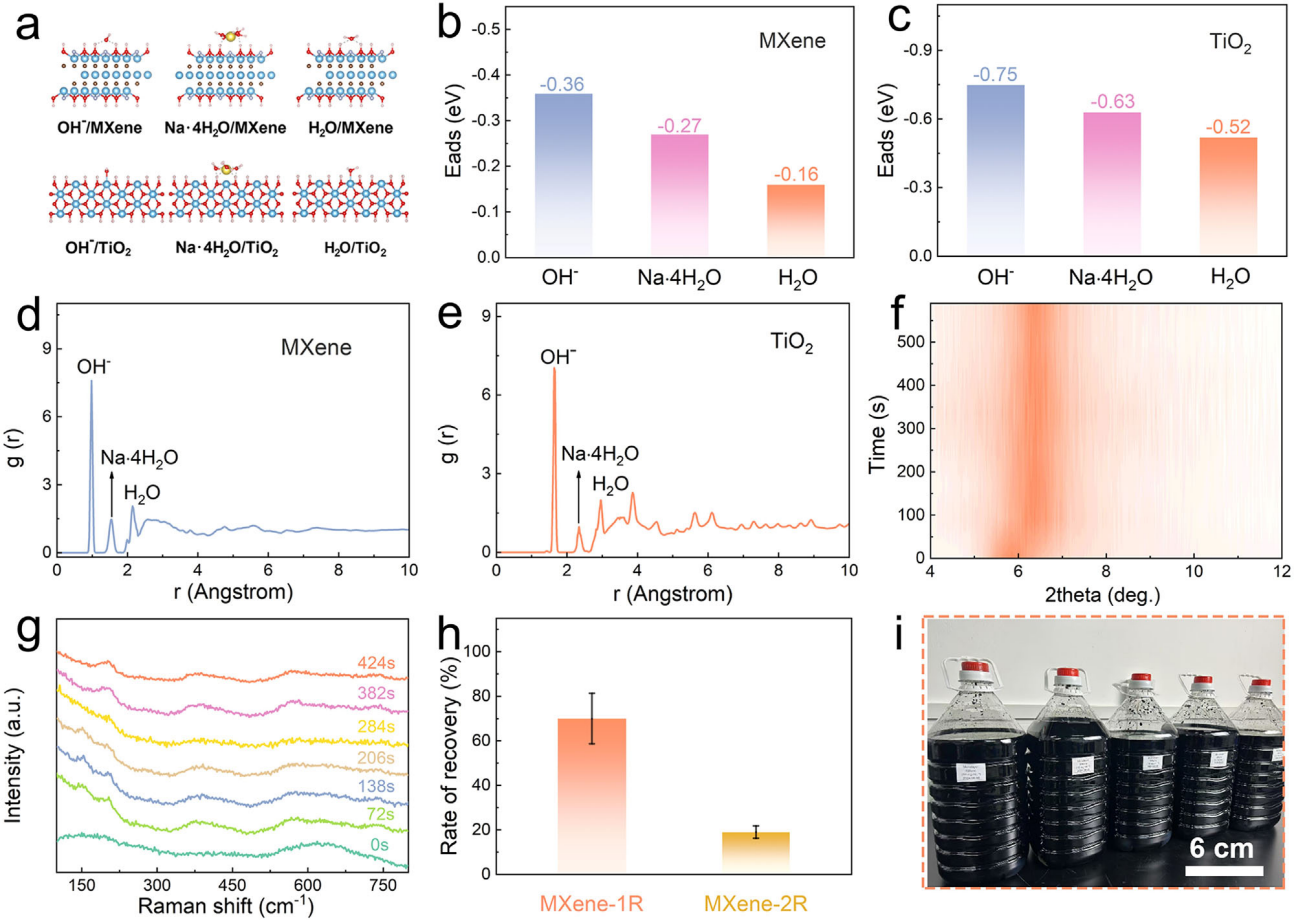
Mechanism and recovery efficiency of the erasure–purification process for oxidized MXene. (a) Side views of adsorption configurations of OH^−^, H_2_O, and Na•4H_2_O on MXene and TiO_2_ surfaces obtained from first‐principles calculations. Corresponding adsorption energies on (b) MXene and (c) TiO_2_ surfaces, respectively. Radial distribution functions (RDFs) of (d) OH^−^, H_2_O, and Na•4H_2_O on MXene and (e) TiO_2_ surfaces derived from molecular dynamics simulations. (f) In situ XRD patterns of MXene‐1L during erasure treatment in 6 M HCl. (g) In situ Raman spectra of MXene‐2L during the erasure process in 6 M HCl. (h) Recovery efficiency of MXene‐1R and MXene‐2R. (i) Scaled‐up erasure and purification of oxidized MXene (∼2 L per bottle).

To clarify the physicochemical origin of surface oxide removal from degraded MXene, density functional theory (DFT) calculations and molecular dynamics (MD) simulations were performed to analyze the interfacial interactions among MXene, ions, and water under alkaline hydrothermal conditions [35]. It should be emphasized that the term ion‐confinement shielding used in this work does not refer to a structurally well‐defined or static interfacial layer, but rather to a dynamically formed, ion‐enriched interfacial environment that limits the accessibility of free water molecules to the MXene surface. In aqueous Ti_3_C_2_T*_x_* dispersions, oxidation of MXene by water proceeds through a multistep pathway involving adsorption of water molecules, dissociation accompanied by hydrogen evolution, extraction of Ti atoms from the lattice, and subsequent attacks on newly exposed Ti sites [19]. These reactions are thermodynamically favored and become more pronounced at elevated temperatures due to the increased reactivity of water, highlighting the necessity of suppressing direct water–surface interactions to mitigate further degradation. DFT calculations (Figure **3**a) reveal that hydroxide ions (OH^−^) and hydrated sodium ions (Na^+^•4H_2_O) exhibit stronger adsorption energies on the negatively charged MXene surface than neutral water molecules (Figure 3b). This preferential adsorption suggests that, under high ionic strength alkaline conditions, hydrated ions are thermodynamically favored to occupy interfacial sites on MXene, thereby reducing the probability of direct water adsorption. Rather than forming a discrete layer, these ions collectively generate a spatially confined interfacial environment that sterically and electrostatically hinders water molecules from approaching the MXene surface. Consistent with the DFT results, MD simulations demonstrate that OH^−^ and Na^+^•4H_2_O preferentially reside within confined regions at approximately 0.98 and 1.51 nm from the MXene surface, respectively, while water molecules display a peak radial distribution at around 2.15 nm (Figure 3d) [17, 36]. This spatial separation indicates that the presence of hydrated ions effectively disrupts the continuous water network near the MXene surface, thereby suppressing water‐mediated oxidation reactions. It should be noted that the MD simulations are intended to capture relative interfacial preferences and dynamic tendencies, rather than to describe a rigid or permanent shielding structure. Experimental evidence further supports the formation of an ion‐confined interfacial environment. Zeta potential measurements of fresh MXene as well as MXene‐1R and MXene‐2R in saturated NaOH solution show a pronounced decrease in absolute values (Figure S20a), consistent with extensive adsorption of positively charged hydrated ions onto the negatively charged MXene surface. Under such high ionic strength conditions, compression of the electrical double layer, together with enhanced electrostatic interactions between partially desolvated Na^+^ ions and MXene, leads to a more compact and dynamically stable interfacial ion distribution, which further limits direct contact between MXene and free water molecules (Figure S20b) [37, 38]. In parallel, OH^−^ ions also exhibit a strong affinity toward TiO_2_ surfaces (Figure 3c,e). During alkaline hydrothermal treatment, these OH^−^ species actively participate in the dissolution and reconstruction of surface‐derived TiO_2_, driving its transformation into sodium titanate intermediates. The concurrent ion‐confinement effects at both the MXene and TiO_2_ interfaces facilitate suppression of further MXene oxidation while enabling efficient conversion of surface oxides into removable titanate species, thereby underpinning the erasure and purification process. In situ characterization further elucidates the kinetics of titanate removal during the subsequent acid‐washing step (Figure 3f). Upon exposure of MXene‐1L to HCl, the interlayer spacing rapidly decreases from 1.98 to 1.38 nm within 100 s and remains stable thereafter. Since sodium titanate is predominantly amorphous and difficult to resolve in the Raman spectrum of MXene‐1L, MXene‐2L was selected for in situ Raman monitoring. As shown in Figure 3g, the characteristic Raman bands of sodium titanate at 276 and 655 cm^−^^1^ gradually weaken and nearly disappear within 284 s, accompanied by the re‐emergence of intrinsic MXene features. These observations confirm that sodium titanate intermediates dissolve rapidly during acid treatment, completing the erasure process within minutes. Collectively, the combined computational and experimental results support a mechanism in which ion‐confinement‐induced interfacial regulation suppresses water‐driven MXene oxidation while promoting the conversion and removal of surface TiO_2_.

### 2.3 Recovery Efficiency of Erasure‐Purified Oxidized MXene

Monolayer dispersions constitute the fundamental form of MXene and provide the basis for constructing various macroscopic MXene assemblies. With continuous advances in synthesis protocols, their scalable production has become increasingly feasible. However, once oxidation occurs, the structure and properties of MXene are progressively altered, such that the material is commonly regarded as unsuitable for further use [12]. Oxidative degradation typically initiates during or shortly after dispersion preparation and proceeds gradually during storage, posing a persistent challenge for reliable performance evaluation and downstream applications. Moreover, many early batches of MXene prepared in research laboratories already exhibit appreciable surface oxidation, further complicating reproducibility and utilization. The erasure‐purification strategy proposed in this work provides an effective route to addresses this issue by enabling the recovery and regeneration of oxidized MXene across different degradation states. Notably, recovery yield represents a key metric for resource recycling and commercial relevance, with direct economic and environmental implications. As shown in Figure 3h, the recovery efficiencies of MXene‐1R and MXene‐2R reach approximately 70% and 19%, respectively, demonstrating the capability of this strategy to reclaim oxidized MXene even after prolonged degradation. When combined with our previously reported semi‐solid‐state MXene processing technology [39, 40], this approach may further improve regeneration efficiency for MXene subjected to long‐term storage. To evaluate scalability, a proof‐of‐concept scale‐up experiment was conducted at the 10 g level (Figure 3i). The MXene nanosheets recovered from large‐volume erasure–purification treatment retained excellent assembly capability and could be reassembled into large‐area freestanding films, exhibiting good mechanical flexibility (Figure S21a) and high electrical conductivity (Figure S21b). These results indicate that the proposed strategy preserves structural integrity and performance reproducibility under scaled‐up processing conditions. Figure S22 compares the process flow of this work with previously reported approaches that show potential for scalable MXene production. In conventional routes, the production cost of MXenes is largely associated with nonferrous metal precursors and the high energy consumption required for MAX phase synthesis and high‐temperature calcination, while environmental burdens primarily arise from the use of hydrofluoric acid or fluoride‐containing etchants. In contrast, the present strategy employs inexpensive and readily available alkaline and acidic reagents and operates at relatively mild reaction temperatures to regenerate and reprocess oxidized MXenes. From a process perspective, this confers advantages in terms of cost control, energy consumption, and environmental compatibility, indicating promising potential for further scale‐up. Furthermore, resource recovery from the waste solutions generated during the acid‐washing step was investigated. During erasure–purification, dissolved Ti^4^^+^ species are mainly retained in the hydrochloric acid waste solution. Upon simple thermal treatment, the gradual reduction in acidity induces spontaneous hydrolysis of Ti ions, leading to the formation of quantum‐sized TiO_2_ nanoparticles. Depending on the storage duration of the degraded MXene, the recovered byproducts are denoted as TiO_2_‐1R and TiO_2_‐2R (Figures S23 and S24). Relative to the initial MXene mass, the yields of TiO_2_‐1R and TiO_2_‐2R reach 23% and 71%, respectively. Consequently, when both regenerated MXene and secondary TiO_2_ quantum dots are considered, the overall recovery efficiency can approach ∼90% or higher. These results suggest that even under severe oxidation conditions, concurrent recovery of secondary products can substantially reduce material waste while enhancing the overall resource utilization efficiency and value of degraded MXene materials.

### 2.4 Universality of the Erasure‐purification Strategy for Oxidized MXene

**FIGURE 4:**
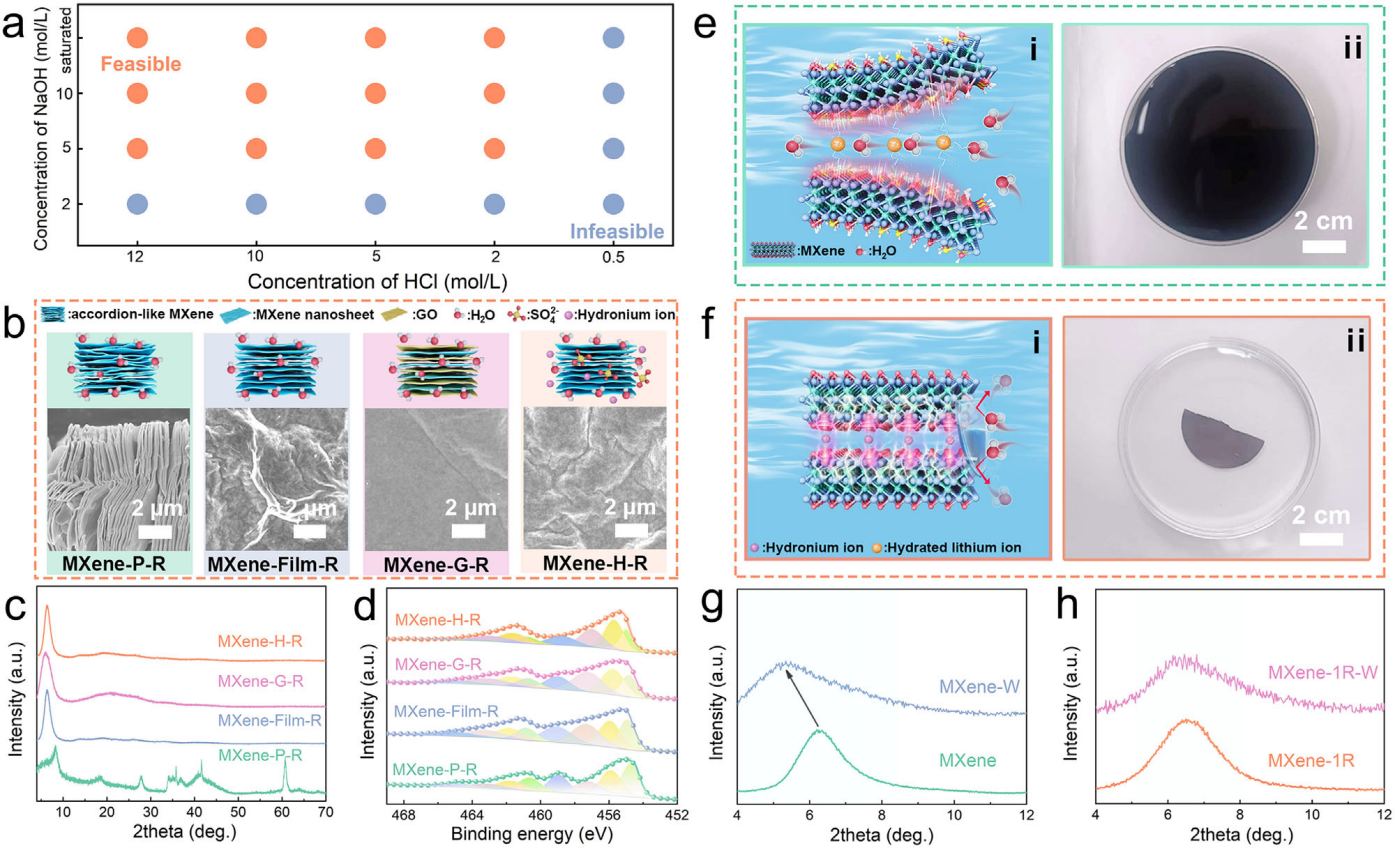
Optimization, universality, and anti‐swelling behavior of the erasure‐purification process for oxidized MXene. (a) Feasibility of the erasure–purification process under different acid and alkali concentrations. (b) SEM images, (c) XRD patterns, and (d) high‐resolution Ti 2p binding energy spectra of oxidized MXene with varied morphologies and storage conditions after purification. Schematic illustration of the anti‐swelling behavior of (e) MXene and (f) MXene‐1R (optical images show the two corresponding films after ultrasonication in deionized water for 10 min). (g,h) XRD patterns of the samples.

During alkaline hydrothermal treatment, preferential adsorption of OH^−^ and hydrated Na^+^ at the MXene‐solution interface generates an ion‐confined interfacial environment that suppresses further oxidation of MXene. It should be noted that this ion‐confinement effect is highly dependent on processing conditions. In particular, insufficient acid concentration during the subsequent washing step can induce hydrolysis of Ti^4^^+^ species, indicating that both alkali and acid concentrations must be carefully optimized to ensure effective purification and recovery. Identifying the minimum effective concentrations of alkali and acid is therefore essential for reducing processing costs and improving operational safety in practical applications. Figures S25 and S26 summarize the surface morphology, phase composition, and Ti chemical states of MXene‐1R films obtained under different NaOH concentrations (10 m, 5 m, and 2 m). When the NaOH concentration is maintained 5 m or higher, the resulting MXene‐1R films exhibit smooth and uniform surfaces (Figure S25a,b), and their phase compositions (Figure S26a) and Ti bonding states (Figure S26b) closely resemble those obtained under saturated alkaline conditions. In contrast, films processed in 2 M NaOH exhibit abundant surface oxide particles (Figure S25c), pronounced TiO_2_ diffraction peaks in XRD patterns, and a high fraction of Ti^4^^+^ species (Figure S26), indicating that the ion‐confinement effect established under low alkali concentration is insufficient to effectively suppress oxidation. These results suggest that the NaOH concentration should be maintained at ≥5 m to ensure reliable oxide conversion and removal. The effect of acid concentration was further evaluated during the erasure step. Experimental results show that an HCl concentration of at least 2 m is required for efficient recovery. When the acid concentration is reduced to 0.5 m, TiO_2_ diffraction peaks are no longer detected in XRD patterns (Figure S27a); however, XPS analysis reveals a pronounced increase in Ti^4^^+^ binding energies components (Figure S27b), and SEM images show that a large number of fibrous nanostructures remain on the film surface (Figure S28). These observations indicate incomplete dissolution and removal of sodium titanate intermediates due to insufficient proton activity, leading to the persistence of amorphous TiO_2_ residues and compromised recovery efficiency. Therefore, effective erasure and reliable regeneration of oxidized MXene require that the working concentrations of alkali and acid be maintained at no less than 5 m and 2 m, respectively (Figure 4a).

To further assess the general applicability of the erasure‐recovery strategy, it was applied to a range of oxidized MXene assemblies prepared under different structural configurations and environmental conditions. These samples included accordion‐like powders obtained via HF etching, stacked films assembled by vacuum filtration, graphene oxide/MXene composite films, and MXene films degraded under simulated sulfuric acid electrolyte exposure. As shown in Figure 4b, surface oxide nanoparticles were effectively removed from all tested samples after erasure treatment. Their phase compositions (Figure 4c) and Ti chemical states (Figure 4d) were restored to levels comparable to those of fresh MXene (Figures S29–S34), indicating that this strategy is applicable across diverse MXene forms and oxidative scenarios. In addition to oxide removal, the erasure‐treated MXene films exhibited a pronounced improvement in resistance to aqueous swelling. Typically, when pristine MXene films are immersed in water, water molecules spontaneously intercalate into the interlayer galleries, leading to lattice expansion and osmotic swelling (Figure 4e‐i) [41]. This process results in a substantial increase in interlayer spacing (Figure 4g) and often causes film delamination or fragmentation under ultrasonication or mechanical agitation (Figure 4e‐ii). In contrast, the interlayer spacing of the erasure‐treated MXene‐1R film remained nearly unchanged under identical immersion conditions (Figure 4h), and no fragmentation was observed after ultrasonication, demonstrating markedly enhanced swelling resistance (Figure 4f). This improved anti‐swelling behavior can be attributed to interlayer ion exchange occurring during the acid washing step. Specifically, the initial co‐intercalation of H^+^ and Li^+^ is replaced by a predominantly single‐ion H^+^ intercalation state, which reduces the effective interlayer spacing. Based on Coulombic considerations, such a reduction in interlayer distance is expected to enhance electrostatic attraction between adjacent MXene layers. Furthermore, our previous studies have shown that hydrated protons, due to their smaller effective ionic radius, can achieve a higher adsorption density on MXene nanosheets [17]. The combined effects of reduced interlayer spacing and enhanced charge screening likely strengthen interlayer interactions, thereby contributing to the observed suppression of swelling. Benefiting from this enhanced resistance to swelling, the erasure–purified MXene maintains structural integrity under demanding conditions, including high humidity, liquid immersion, and ultrasonic agitation. This robustness provides clear advantages for maintaining the structural and functional stability of MXene assemblies in moisture‐rich or mechanically perturbed environments.

### 2.5 Application Potential of Erasure‐Recovered MXene

**FIGURE 5:**
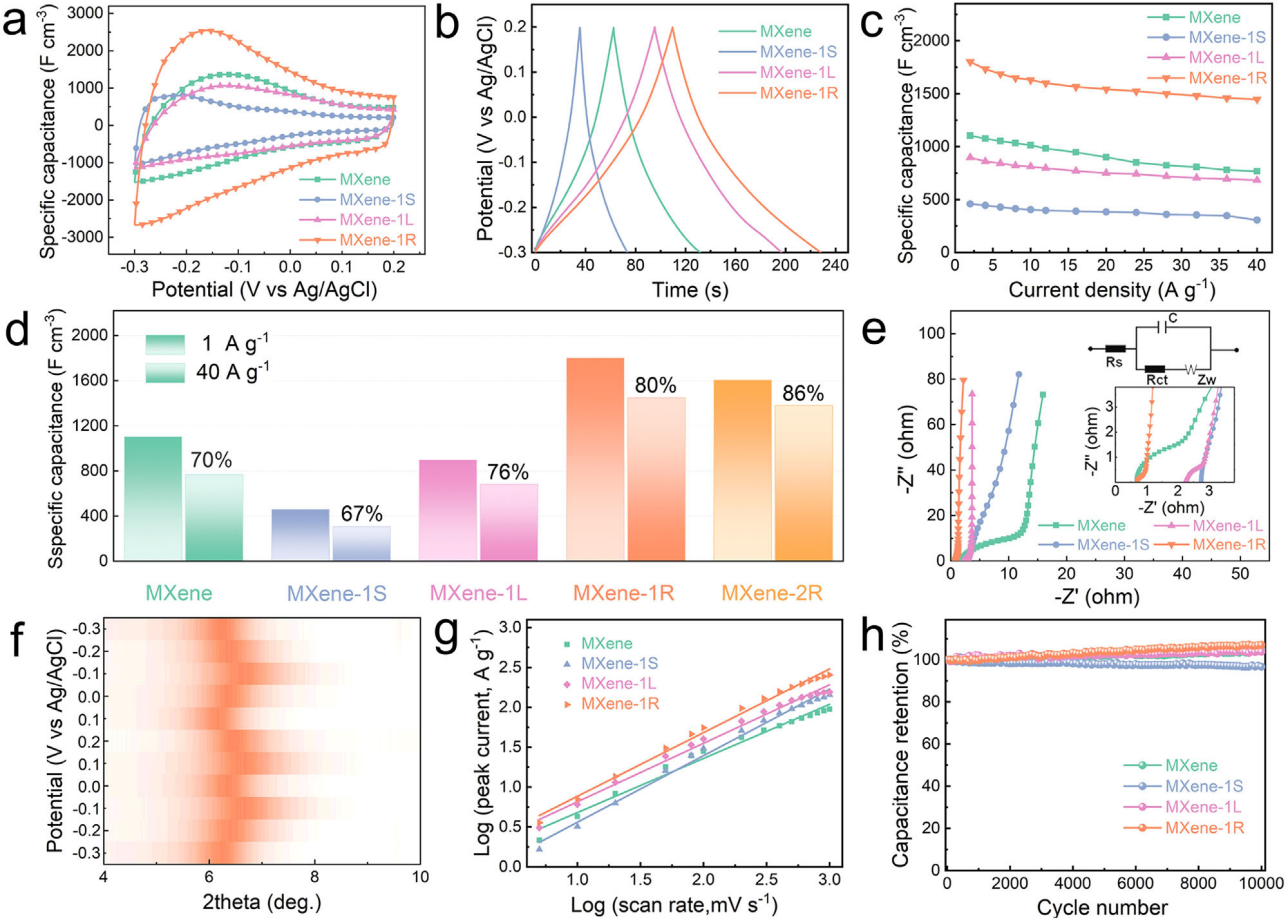
Electrochemical energy storage performance of MXene after erasure–purification. (a) CV curves at a scan rate of 50 mV s^−^^1^ and (b) GCD curves at a current density of 2 A g^−^^1^ for fresh MXene, MXene‐1S, MXene‐1L, and MXene‐1R. (c) Specific capacitance at different current densities and (d) corresponding rate performance. (e) Nyquist plots obtained from electrochemical impedance spectroscopy (EIS). (f) Evolution of the (002) diffraction peak of MXene‐1R during charge–discharge cycling. (g) Relationship between peak current and scan rate for each sample. (h) Cycling stability of MXene, MXene‐1S, MXene‐1L, and MXene‐1R over 10 000 cycles.

To evaluate the practical applicability of the recovered MXene, the electrochemical performance of MXene‐1R, MXene‐1S, MXene‐1L, and fresh MXene was investigated using supercapacitor configurations. As shown in Figure 5a, all samples exhibit quasi‐rectangular cyclic voltammetry (CV) curves, characteristic of combined electric double‐layer capacitance and pseudocapacitance behavior [42, 43, 44, 45]. Among them, MXene‐1R displays the largest enclosed area (Figure 5a and Figure S35), indicating an enhanced charge‐storage capability. This improvement can be attributed to the erasure–purification process, which removes electrochemically inactive surface TiO_2_ particles and sodium titanate nanowires, while concurrently converting halogen‐terminated surface groups into oxygen‐containing functionalities that contribute to pseudocapacitance [33, 46, 47]. The superiority of MXene‐1R is further confirmed by galvanostatic charge–discharge (GCD) measurements (Figure 5b and Figure S36). In addition, the densely reconstructed lamellar architecture of the recovered MXene film contributes to its volumetric capacitance. As shown in Figure 5c, MXene‐1R delivers a volumetric capacitance of 1801 F cm^−^^3^ at 1 A g^−^^1^, ranking among the highest values reported for MXene‐based electrodes (Table S1). This value corresponds to a 1.6‐fold increase relative to fresh MXene (1103 F cm^−^^3^), a 2.0‐fold increase compared with MXene‐1L (898 F cm^−^^3^), and a 3.9‐fold increase over MXene‐1S (459 F cm^−^^3^). It is worth noting that the removal of surface TiO_2_ generates nanoscale pore defects, which in combination with surface oxygen functionalization, offer more active sites for redox reactions and reduce ion diffusion resistance. As shown in Figure 5d, when the current density was increased to 40 A g^−^^1^, MXene‐1R retained 80% of its initial volumetric capacitance (1448 F cm^−^^3^), while MXene‐2R exhibited an even higher retention of 86% (1380 F cm^−^^3^) (Figure S37), demonstrating excellent rate capability of the recovered MXene. Electrochemical impedance spectroscopy (EIS) results are shown in Figure 5e. Owing to the intrinsically high electrical conductivity of MXene and the preservation of a continuous conductive network in MXene‐1R, both fresh MXene and MXene‐1R exhibit similarly low solution resistance (R_s_). In contrast, MXene‐1S and MXene‐1L show substantially increased R_s_ values due to partial disruption of conductive pathways by insulating TiO_2_ and sodium titanate phases [48, 49]. The charge‐transfer resistance (R_ct_), reflected by the semicircle diameter in the Nyquist plots, is lowest for MXene‐1R, indicating more favorable interfacial charge‐transfer kinetics and improved electrolyte ion accessibility. This enhancement likely arises from the introduction of oxygen‐containing surface terminations, which provide additional reversible redox‐active sites, as well as improved electrolyte wettability. MXene‐1R also exhibits reduced ion diffusion resistance, which can be attributed to the combined effects of enhanced surface hydrophilicity, shortened ion‐transport pathways resulting from reduced nanosheet dimensions after redispersion, and improved interlayer accessibility [50, 51]. In situ XRD measurements (Figure 5f) reveal that the interlayer spacing of MXene‐1R varies by only ∼0.11 nm during charge–discharge cycling. The preferential substitution of interlayer species by protons effectively suppresses spontaneous water intercalation while still allowing reversible proton insertion and surface protonation of oxygen‐containing groups, thereby contributing to enhanced pseudocapacitive behavior. To further probe charge storage dynamics, we analyzed the relationship between scan rate (*v*) and peak current. The b‐value for MXene‐1R was determined to be 0.80, higher than that of fresh MXene (0.68) (Figure 5g), suggesting a more surface‐controlled capacitive storage mechanism and superior kinetic properties. Notably, MXene‐1R exhibits outstanding cycling stability, retaining approximately 107% of its initial capacitance after 10 000 charge–discharge cycles (Figure 5h). This anomalous increase in capacitance may be attributed to the repeated intercalation and deintercalation of electrolyte ions during prolonged cycling, which gradually enlarges the interlayer spacing of MXene nanosheets (Figure S38) and thereby enhances electrolyte ion accessibility to electrochemically active sites. These results demonstrate that the proposed erasure–purification strategy not only enables functional recovery of degraded MXene but also markedly improves its structural and electrochemical cycling stability. When assembled into symmetric supercapacitor devices, MXene‐1R also demonstrates superior performance, as evidenced by its CV and GCD profiles (Figures S39 and S40). Specifically, at a power density of 1980 mW cm^−^^3^, MXene‐1R achieved an energy density of 33.0 mWh cm^−^^3^, outperforming fresh MXene (16.6 mWh cm^−^^3^), MXene‐1L (12.6 mWh cm^−^^3^), and MXene‐1S (9.1 mWh cm^−^^3^). MXene‐2R also exhibited excellent electrochemical performance, delivering an energy density of 30.5 mWh cm^−^^3^, which surpasses that of most previously reported MXene‐based symmetric devices (Figure S41) [52, 53, 54]. The superior electrochemical performance of the erasure–purified MXene primarily arises from the synergistic interplay between its structural characteristics and surface chemical modulation. Specifically, i) the introduction of oxygen‐containing functional groups provides additional reversible redox‐active sites for electrochemical reactions; ii) the reduced nanosheet dimensions significantly shorten electrolyte ion diffusion pathways, thereby facilitating rapid charge transport; and iii) the densely reconstructed lamellar architecture endows the material with a high volumetric density advantage. Collectively, these results demonstrate the substantial potential of erasure‐recovered MXene for high‐performance electrochemical energy storage applications.

**FIGURE 6:**
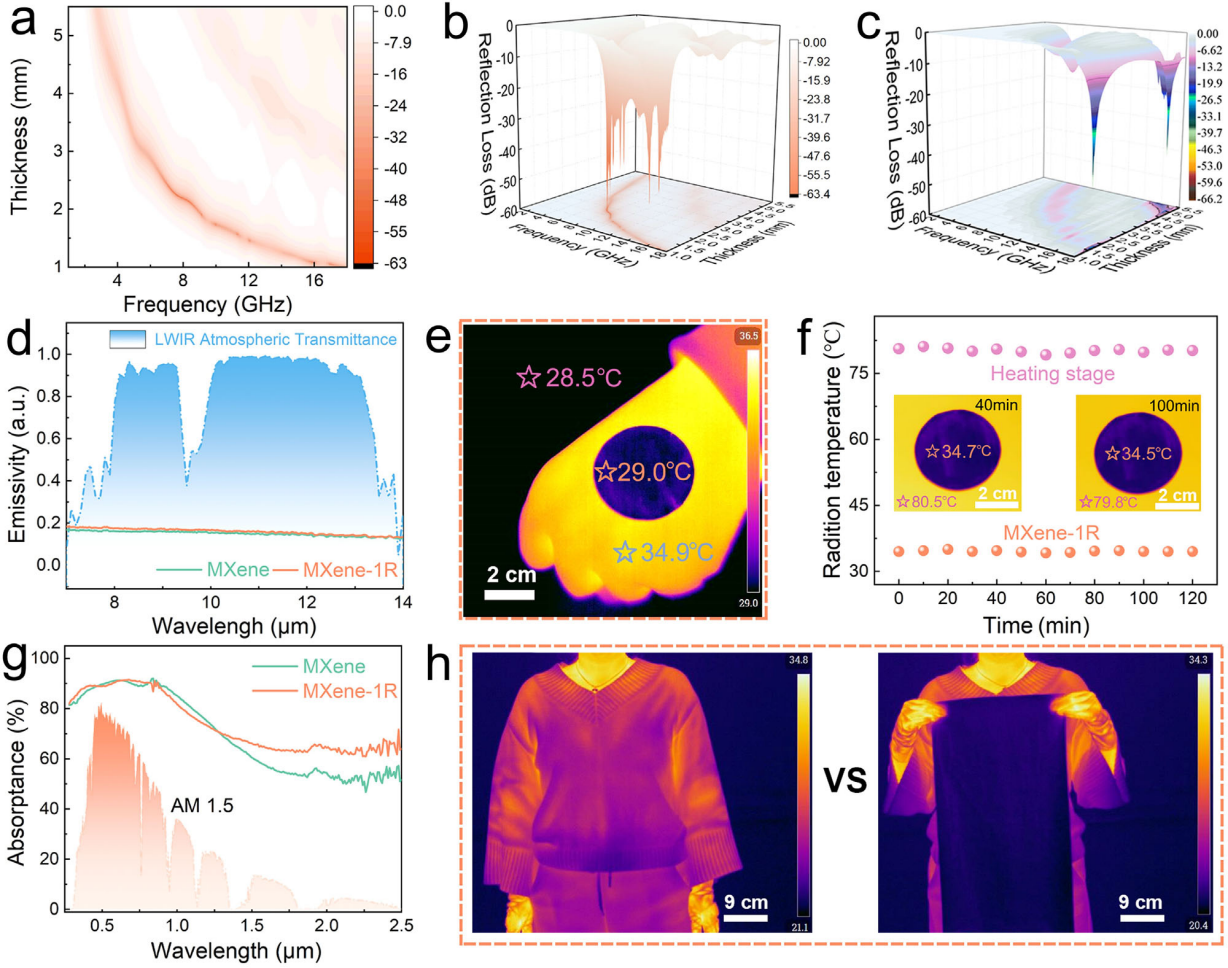
Electromagnetic wave absorption and infrared stealth performance of MXene after erasure–purification. (a) Planar view of the EM RL of MXene‐1R. 3D plot of RL values of (b) MXene‐1R and (c) MXene. (d) Comparison of infrared emissivity between MXene and MXene‐1R. (e) Infrared image of an MXene‐1R film placed on a human fist. (f) Infrared stealth performance of an MXene‐1R film continuously placed on a heating stage at 80°C. (g) Planar views of the EM RL of MXene and MXene‐1R. (h) Infrared radiation demonstration of a fabric (thickness: 2 µm) coated with MXene‐1R dispersion when placed over the human body.

As MXene materials move toward practical and large‐scale applications, identifying functions with clear performance advantages and application relevance becomes increasingly important [55, 56]. The erasure‐recovered MXene (MXene‐1R) developed in this work combines high electrical conductivity with abundant surface‐active sites, making it a versatile candidate not only for electrochemical energy storage but also for electromagnetic wave absorption and infrared stealth applications [57]. To evaluate its electromagnetic absorption performance, the MXene‐1R dispersion was converted into a low‐density powder (∼0.2 g cm^−^^3^) via freeze‐drying. As shown in Figure 6a,b, the minimum reflection loss (RL_min_) of the MXene‐1R powder reaches −53.4 dB at 16 GHz, outperforming that of fresh MXene powder, which exhibits an RL_min_ of −50 dB at 14 GHz (Figure 6c and Figure S42). Notably, MXene‐1R also demonstrates effective electromagnetic wave attenuation across the low‐frequency range of 2.0–8.0 GHz, which is highly relevant to electronic devices. In particular, this absorption band fully covers the n79 frequency band (4.4–5.0 GHz) used in 5G communications, whereas fresh MXene shows limited attenuation in this region. Achieving broadband and efficient low‐frequency electromagnetic absorption in a single‐component material remains challenging for many reported systems. The enhanced absorption performance of MXene‐1R can be attributed to the cooperative contribution of multiple attenuation mechanisms. Specifically, the recovered MXene retains a continuous conductive network, enabling pronounced conductive loss. Meanwhile, surface functionalization, the introduction of in‐plane defects, and the formation of hierarchical interfacial features collectively strengthen dipolar polarization and interfacial polarization losses. In addition, the reduced nanosheet dimensions increase the density of internal interfaces, facilitating multiple reflection and scattering processes, which together contribute to more effective electromagnetic wave attenuation, particularly in the low‐frequency region [58, 59, 60, 61]. The combined action of these mechanisms improves impedance matching and promotes efficient dissipation of incident electromagnetic radiation. Beyond performance enhancement, these results also provide a different perspective on oxidation‐related degradation in MXene dispersions. Rather than being solely detrimental, surface oxidation products can be selectively transformed or removed through targeted erasure treatments, enabling recovery and even improvement of multifunctional properties. Importantly, MXene dispersions can be stored under ambient conditions without energy‐intensive preservation, and their functional characteristics can be tuned post‐storage through the erasure–purification protocol. This processing flexibility enhances material utilization efficiency and offers practical advantages for future deployment of MXene‐based technologies [62].

In the domain of infrared stealth and thermal management, MXene‐1R also demonstrates exceptional performance. In the mid‐to‐far infrared region, the MXene‐1R film exhibits a low average emissivity of approximately 0.15 (Figure 6d), comparable to that of pristine MXene. As shown in Figure 6e, when an MXene‐1R film is placed over a human fist, the apparent surface temperature in infrared imaging becomes nearly indistinguishable from the surrounding background, producing an effective infrared camouflage effect. Notably, this infrared concealment remains stable over a broad temperature range. MXene‐1R maintains reliable radiative thermal masking under sustained heating at 40°C (Figure S43a), 80°C (Figure 6f), 120°C (Figure S43b), and even at temperatures up to 240°C (Figure S43c), indicating robust infrared stealth capability across practical operating conditions [63, 64, 65, 66]. This behavior primarily arises from the densely packed and continuous lamellar architecture formed upon reassembly, which endows the film with a high carrier concentration and a metallic electromagnetic response. These characteristics effectively suppress outward infrared radiation and reduce the apparent infrared signature. In addition to low infrared emissivity, MXene‐1R exhibits a high solar absorptivity of up to 84.6% (Figure 6g), suggesting its potential utility for solar‐assisted thermal management in outdoor environments. Benefiting from this combination of properties, MXene‐1R can be readily integrated into textile substrates. For instance, even ultra‐thin fabrics (∼2 µm) can be functionalized by simple dip‐coating with MXene‐1R dispersions, imparting both low infrared emissivity and high solar absorptivity. Such MXene‐coated fabrics can efficiently harvest solar radiation to achieve rapid warming under cold outdoor conditions, while simultaneously suppressing excessive radiative heat loss through atmospheric transmission windows (Figure 6h). Infrared imaging further confirms that these fabrics significantly reduce radiative heat dissipation from the human body when worn, indicating enhanced passive thermal insulation performance. Owing to this dual mechanism of solar energy absorption and infrared radiation suppression, MXene‐1R‐coated textiles show promise for use as outer‐layer materials in applications such as outdoor sportswear, cold‐weather protective gear, and advanced thermal insulation systems. Looking forward, integration of MXene‐based infrared‐stealth materials with emerging radiative cooling concepts could enable bidirectional thermal regulation, offering adaptability across varying environmental conditions. While such multifunctional systems remain a subject for future exploration, the present results demonstrate that oxide erasure–purification provides an effective pathway for restoring and enhancing MXene performance, while simultaneously expanding its applicability across electromagnetic attenuation, infrared stealth, thermal management, and textile‐based platforms.

## 3 Conclusions

In summary, this work presents an ion‐confinement–assisted erasure‐purification strategy to addresses oxidation‐induced degradation of MXene in aqueous environments. By integrating alkaline hydrothermal treatment with a subsequent acid‐washing step, surface‐derived oxide species are selectively transformed and removed, enabling effective regeneration of oxidized MXene under mild conditions. The recovered MXene exhibits restored and tunable surface chemistry, reconstructed lamellar architectures, and enhanced multifunctional performance, including electrochemical energy storage, electromagnetic wave absorption, and infrared stealth. The strategy is applicable across different MXene morphologies, oxidation states, and assembly forms, and remains effective under scaled‐up processing conditions. Beyond material recovery, this work provides new insight into the interfacial chemistry and structural evolution of MXene during early‐stage oxidation and regeneration. By reframing oxidation as a modifiable and partially recoverable process, the proposed approach expands the design space for MXene recycling, reprocessing, and functional optimization, supporting its broader technological development.

## 4 Experimental Section

### 4.1 Synthesis of Monolayer Ti_3_C_2_T_x_ MXene Dispersion

Monolayer Ti_3_C_2_T_x_ nanosheets were synthesized using a modified minimally intensive layer delamination (MILD) etching method.[10, 67, 68, 69] Briefly, 4 g of LiF (Aladdin) was dissolved in 80 mL of 12 m. HCl (Xilong Chemical) under continuous stirring. Subsequently, 4 g of Ti_3_AlC_2_ powder (Harbin Zhisu Future Technology Co., Ltd.) was gradually added to the solution, and the mixture was stirred at 45°C and 200 rpm for 48 h to complete the etching reaction. After cooling to room temperature, the resulting suspension was centrifuged and repeatedly washed with deionized water until the supernatant reached neutral pH. The obtained sediment, exhibiting a clay‐like appearance, was then redispersed in 500 mL of deionized water and ultrasonicated in an ice bath for 1 h to facilitate delamination. The exfoliated suspension was centrifuged at 3600 rpm for 5 min to remove unexfoliated particles. This centrifugation step was repeated four times to ensure effective separation of monolayer Ti_3_C_2_T*_x_* nanosheets. The nominal concentration of the obtained MXene dispersion was determined by vacuum filtration. Specifically, 5 mL of the supernatant was filtered through a cellulose membrane and dried at 65°C for 12 h. The dried mass was then used to calculate the nominal concentration of the monolayer Ti_3_C_2_T_x_ MXene dispersion.

### 4.2 Oxidation Simulation and Erasure‐Purification of Ti_3_C_2_T_x_ MXene

The as‐prepared Ti_3_C_2_T*_x_* MXene dispersion was diluted with deionized water to a concentration of 2 mg mL^−^^1^ and sealed in 20 mL polyethylene bottles. The dispersions were stored at 25°C for 1 and 2 months to simulate oxidative degradation, yielding samples denoted as MXene‐1S and MXene‐2S, respectively. To initiate the erasure–purification process, a small amount of concentrated HCl (two drops) was added to each aged dispersion, followed by centrifugation at 6000 rpm to collect the sediment. Saturated NaOH solution at room temperature was then added to the sediment, which was thoroughly stirred and transferred into a 20 mL Teflon‐lined stainless‐steel autoclave for hydrothermal treatment at 120°C for 4 h. After naturally cooling to room temperature, the reaction product was centrifuged at 8000 rpm. The collected solid was repeatedly washed with deionized water until a neutral pH was achieved and subsequently redispersed in deionized water by ultrasonication for 10 min, yielding an intermediate sodium titanate/Ti_3_C_2_T*_x_* composite suspension, denoted as MXene‐1L and MXene‐2L. Subsequently, concentrated HCl cooled to 6–8°C was directly added to the hydrothermally treated precipitate. The suspension was stirred at room temperature for 5 min and centrifuged to collect the sediment. This acid‐washing process was repeated four times. The resulting solid was washed with deionized water until neutral and redispersed by ultrasonication, producing the erasure‐purified Ti_3_C_2_T*_x_* MXene dispersions, denoted as MXene‐1R and MXene‐2R. For consistency, all MXene dispersions used in this work were adjusted to a concentration of 2 mg mL^−^^1^. Freestanding MXene films were fabricated by vacuum filtration using circular membranes with a radius of 2.1 cm, with a mass loading of approximately 30 mg.

### 4.3 Preparation and Recovery of TiO_2_ Quantum Dots byproducts

The supernatant collected after the initial acid‐washing step was transferred to a round‐bottom flask and evaporated to dryness at 80°C. The resulting residue was then purified by dialysis to remove residual ionic species, followed by drying at 65°C for 12 h to obtain the TiO_2_ quantum dot byproducts. Depending on the storage duration of the degraded MXene precursor, the recovered products were denoted as TiO_2_‐1R and TiO_2_‐2R, respectively.

### 4.4 Universality of Erasure and Purification for Oxidized Ti_3_C_2_T_x_ MXene

Accordion‐like Ti_3_C_2_T*_x_* powder (denoted as MXene‐P) was prepared by gradually adding 2 g of Ti_3_AlC_2_ precursor into 80 mL of HF (≥40%, Aladdin) under continuous stirring at 65°C for 5 h. The resulting suspension was centrifuged and washed repeatedly washed with deionized water until a neutral pH was reached, followed by dried at 65°C for 12 h to obtain accordion‐structured Ti_3_C_2_T*_x_* powder. A freestanding Ti_3_C_2_T*_x_* film (MXene‐Film) was fabricated by vacuum‐assisted filtration. For preparation of the GO/Ti_3_C_2_T*_x_* composite film (MXene‐G), 3 mg of graphene oxide (GO) suspension (Suzhou Xianfeng Nano Technology Co., Ltd) was added to a Ti_3_C_2_T*_x_* dispersion containing 27 mg of nanosheets. The mixture was thoroughly stirred, vacuum‐filtered, air‐dried at ambient conditions for 4 h, and further dried at 65°C for 12 h to obtain a self‐supporting composite film. MXene‐P (200 mg), Ti_3_C_2_T*_x_*‐Film, and MXene‐G were each sealed in deionized water and stored at 25°C for 3 months, 12 days, and 12 days, respectively. After storage, the water was removed, and the samples were dried at 65°C for 12 h, resulting in oxidized analogs denoted as MXene‐P‐S, MXene‐Film‐S, and MXene‐G‐S. These oxidized samples were subjected to the same erasure and purification procedures as described above, and the resulting purified materials were named MXene‐P‐R, MXene‐Film‐R, and MXene‐G‐R. To simulate degradation and recovery in electrolyte environments, the stacked MXene film was immersed in 3 m H_2_SO_4_ and stored in a sealed container at room temperature for 15 days. After washing with deionized water to neutrality and drying, the resulting oxidized film was denoted as MXene‐H‐S. The stored sample was then purified following the same erasure strategy, and the final regenerated product was designated as MXene‐H‐R.

### 4.5 Characterization Methods

The crystalline phases of the samples were analyzed using an Ultima IV X‐ray diffractometer (Rigaku, Japan) equipped with Cu Kα radiation (λ = 0.154 nm), operating over a scan range of 4°–70°. The surface morphology and elemental composition were examined using a scanning electron microscope (SEM, Hitachi SU‐8010) equipped with an energy‐dispersive X‐ray spectroscopy (EDS, EDAX) detector. Transmission electron microscopy (TEM) was performed on a JEM‐2100F and FEI TALOS.F200X to assess the microstructure and elemental distribution. The chemical binding states of elements were determined using X‐ray photoelectron spectroscopy (XPS, ESCALAB 250Xi, Thermo Fisher Scientific) with Al Kα radiation (hv = 1486.6 eV) under a 15 kV acceleration voltage. Prior to XPS measurement, all film samples were sputter‐cleaned with Ar^+^ ions for 60 s. The zeta potential of nanosheet dispersions was measured using a Horiba SZ‐100 nanoparticle analyzer. The surface functional groups were identified via Fourier‐transform infrared spectroscopy (FTIR, Nicolet Nexus 670). Electrical conductivity was measured using a four‐point probe system (HPS2662, Helpass Electronic Technology Co., Ltd., Hangzhou, China). The thickness of the monolayer nanosheets was determined via atomic force microscopy (AFM, Dimension Fastscan). Raman spectroscopy was performed using an inVia Raman spectrometer (Renishaw) with an excitation wavelength of 532 nm. To minimize laser‐induced oxidation of MXene during measurement, the laser power was carefully controlled at 2.5 mW. UV–vis absorption spectra were recorded using a UV‐2600i spectrophotometer over a wavelength range of 350–1000 nm. Samples were measured in a quartz cuvette with an optical path length of 1 cm, and all dispersions were diluted by a factor of 150 prior to measurement to ensure consistency. In situ XRD during acid washing (Figure 3f) was conducted on the Rigaku Ultima IV instrument over the 4°–12° range. A ∼1 × 1 cm^2^ piece of vacuum‐filtered MXene‐1L film was placed flat on a glass sample holder, and 6 m HCl was dropped onto the film surface until fully wetted. Continuous measurements were then immediately carried out. In situ Raman spectroscopy during acid washing (Figure 3g) followed a similar procedure. MXene‐2L powder was immobilized on the sample stage, and 6 m HCl was applied. Rapid focusing was performed prior to continuous spectral acquisition. In situ electrochemical XRD tests were conducted on a Bruker D8 diffractometer using 3 m H_2_SO_4_ as the electrolyte. MXene‐1R served as the working electrode, with Ag/AgCl and Pt foil as the reference and counter electrodes, respectively. Measurements were performed over a potential range of −0.3–0.2 V at a scan rate of 5 mV s^−^^1^, and a 4°–12° diffraction angle range was used. Solar reflectance spectra of different MXene samples were recorded using a Lambda 950 UV–vis–NIR spectrophotometer (PerkinElmer, USA) equipped with an integrating sphere (150 mm diameter). Infrared emissivity in the 8–13 µm atmospheric window was measured using a Nicolet iS50 FT‐IR spectrometer (Thermo Fisher) equipped with a gold integrating sphere (model 660–107400). Electrochemical measurements were performed in a Swagelok‐type three‐electrode configuration using 3 m H_2_SO_4_ as the electrolyte. Freestanding MXene films with a diameter of approximately 3 mm were used as the working electrode. A platinum foil and an Ag/AgCl electrode were employed as the counter and reference electrodes, respectively. All electrochemical tests were conducted using a CHI 760e electrochemical workstation (Chenhua Instruments, Shanghai, China). The electrochemical performances of the erasure‐purified MXene samples were systematically evaluated through cyclic voltammetry (CV), galvanostatic charge–discharge (GCD), electrochemical impedance spectroscopy (EIS), and cycling stability tests.

### 4.6 Ethics Statement and Informed Consent

The infrared imaging experiment involving a human hand shown in Figure 6e was conducted solely for demonstration purposes to visualize the infrared stealth performance of the material. The hand served only as a passive heat source. No clinical, diagnostic, or biomedical tests were performed, and no personal identifying information was collected. Written informed consent was obtained from the participant prior to the experiment. According to the relevant institutional guidelines, this type of non‐medical, non‐biological sampling demonstration experiment does not require approval from an ethics committee.

### 4.7 Computational Methods

Density functional theory (DFT) calculations were performed using the Vienna Ab initio Simulation Package (VASP) [70]. The interaction between ions and electrons was described using the projector augmented wave (PAW) method, and the exchange–correlation functional was treated within the Perdew–Burke–Ernzerhof (PBE) generalized gradient approximation [71, 72]. A plane‐wave basis set with a cutoff energy of 500 eV was adopted. Geometry optimizations were carried out until the residual force on each atom was below 0.01 eV/Å and the total energy change between two consecutive steps was less than 10^−^^5^ eV. The Ti_3_C_2_T*_x_* MXene model was constructed by cleaving the Ti_3_AlC_2_ MAX phase along the (0001) plane and removing the Al layer, followed by full surface termination with F, and OH functional groups, which are commonly reported for aqueous‐processed Ti_3_C_2_T*_x_* MXene. A 2 × 2 × 1 supercell was adopted, with a vacuum layer of 20 Å along the surface normal direction to eliminate interactions between periodic images. The surface functional groups were randomly distributed with uniform coverage, and edge atoms were saturated with H atoms to avoid artificial dangling bonds. Brillouin zone sampling was performed using a Monkhorst–Pack k‐point mesh of 3 × 3 × 1. Spin polarization was not considered. During geometry optimization, the bottom Ti layers were fixed to mimic bulk‐like behavior, while the surface Ti atoms, surface functional groups, and adsorbed species were allowed to relax freely. For systems involving aqueous environments, solvation effects were considered using an implicit solvation model (VASPsol) with a dielectric constant of 78.5, corresponding to liquid water at room temperature. The binding energy (E_b_) was calculated using the formula​

(1) Eb=Etotal−i∑Ei,

where E_total​_is the total energy of the MXene–adsorbate composite system and Ei represent the energies of the isolated MXene slab and individual adsorbates calculated under the same computational conditions. The binding energy analysis was used for relative comparison of adsorption preferences among different species, rather than for absolute thermodynamic predictions.

Classical molecular dynamics (MD) simulations were performed using the Forcite module in Materials Studio. The COMPASSII force field was employed to describe the interatomic interactions [73]. The MXene model used in MD simulations was consistent with the DFT‐optimized structure, including F/OH surface terminations and H‐saturated edges. The MXene slab was immersed in an explicit aqueous environment constructed using a cubic water box filled with TIP3P water molecules, with the water density maintained at approximately 1 g cm^−^^3^. MD simulations were carried out using an initial NVT equilibration at 300 K, followed by an NPT production run at 300 K and 1 atm. A time step of 1 fs was used, and the total simulation time was 200 ps. Temperature and pressure were controlled using standard thermostat and barostat algorithms, and the equations of motion were integrated using the velocity Verlet algorithm. During MD simulations, the Ti_3_C_2_ MXene backbone was treated as rigid to focus on the interfacial behavior of surface functional groups, ions, and water molecules. Interfacial water and ion distributions were analyzed based on time‐averaged structural statistics to evaluate local ion enrichment and water organization near the MXene surface.

## Conflicts of Interest

The authors declare no conflicts of interest.

## Supporting information




**Supporting File**: advs73995‐sup‐0001‐SuppMat.docx.

## Data Availability

The data that support the findings of this study are available from the corresponding author upon reasonable request.

## References

[1] M. Naguib , M. W. Barsoum , and Y. Gogotsi , “Ten Years of Progress in the Synthesis and Development of MXenes,” Advanced Materials 33 (2021): 2103393, 10.1002/adma.202103393. 34396592

[2] A. VahidMohammadi , J. Rosen , and Y. Gogotsi , “The World of Two‐dimensional Carbides and Nitrides (MXenes),” Science 372 (2021): 1581, 10.1126/science.abf1581. 34112665

[3] T. Y. Ko , H. Ye , G. Murali , et al., “Functionalized MXene ink Enables Environmentally Stable Printed Electronics,” Nature Communication 15 (2024): 3459. 10.1038/s41467-024-47700-y PMC11043420 38658566

[4] J. Yang , M. Li , S. Fang , et al., “Water‐induced Strong Isotropic MXene‐bridged Graphene Sheets for Electrochemical Energy Storage,” Science 383 (2024): 771–777, 10.1126/science.adj3549. 38359121

[5] A. Sarycheva , A. Polemi , Y. Liu , K. Dandekar , B. Anasori , and Y. Gogotsi , “2D Titanium Carbide (MXene) for Wireless Communication,” Science Advances 4 (2018): 0920, 10.1126/sciadv.aau0920. PMC6155117 30255151

[6] R. Li , Y. Huangfu , L. Liu , et al., “Intercalation‐Induced Interlayer and Defect Engineering in Ti_3_C_2_T* _x_ * MXene for Ultralow‐Reflection Electromagnetic Interference Shielding,” ACS Nano 19 (2025): 2777–2787, 10.1021/acsnano.4c15343. 39772480

[7] K. K. Kazemi , E. Hosseini , S. Hu , et al., “MXene Membrane in Planar Microwave Resonant Structures for 5G Applications,” Applied Materials Today 26 (2022): 101294.

[8] X. Xu , T. Guo , M. Lanza , and H. N. Alshareef , “Status and Prospects of MXene‐based Nanoelectronic Devices,” Matter 6 (2023): 800–837.

[9] C. E. Shuck , A. Sarycheva , M. Anayee , et al., “Scalable Synthesis of Ti_3_C_2_T_x_ MXene,” Advanced Engineering Materials 22 (2020): 1901241, 10.1002/adem.201901241.

[10] X. Shi , Z. Yu , Z. Liu , et al., “Scalable, High‐Yield Monolayer MXene Preparation from Multilayer MXene for Many Applications,” Angewandte Chemie International Edition 64 (2025): 202418420, 10.1002/anie.202418420. 39401092

[11] C. E. Shuck and Y. Gogotsi , “Taking MXenes from the Lab to Commercial Products,” Chemical Engineering Journal 401 (2020): 125786, 10.1016/j.cej.2020.125786.

[12] M. Shekhirev , C. E. Shuck , A. Sarycheva , and Y. Gogotsi , “Characterization of MXenes at every Step, from Their Precursors to Single Flakes and Assembled Films,” Progress in Materials Science 120 (2021): 100757, 10.1016/j.pmatsci.2020.100757.

[13] T. S. Mathis , K. Maleski , A. Goad , et al., “Modified MAX Phase Synthesis for Environmentally Stable and Highly Conductive Ti_3_C_2_ MXene,” ACS Nano 15 (2021): 6420–6429, 10.1021/acsnano.0c08357. 33848136

[14] H. Shi , P. Zhang , Z. Liu , et al., “Ambient‐Stable Two‐Dimensional Titanium Carbide (MXene) Enabled by Iodine Etching,” Angew Chem Int Ed 60, 8689–8693 (2021). 10.1002/anie.202015627 PMC8048443 33484049

[15] C. J. Zhang , S. Pinilla , N. McEvoy , et al., “Oxidation Stability of Colloidal Two‐Dimensional Titanium Carbides (MXenes),” Chemistry of Materials 29 (2017): 4848–4856, 10.1021/acs.chemmater.7b00745.

[16] X. Zhao , A. Vashisth , E. Prehn , et al., “Antioxidants Unlock Shelf‐Stable Ti_3_C_2_T* _x_ * (MXene) Nanosheet Dispersions,” Matter 1 (2019): 513–526.

[17] X. Zhang , X. Liu , Y. Feng , et al., “Stabilizing the MXene by Ion Confinement Shielding in a Wide Temperature Range,” Small Structure 4 (2023): 2200309.

[18] P. Hou , Y. Tian , Y. Xie , et al., “Unraveling the Oxidation Behaviors of MXenes in Aqueous Systems by Active‐Learning‐Potential Molecular‐Dynamics Simulation,” Angewandte Chemie International Edition 62 (2023): 202304205, 10.1002/anie.202304205. 37313787

[19] T. Wu , P. R. C. Kent , Y. Gogotsi , and D.‐E. Jiang , “How Water Attacks MXene,” Chemistry of Materials 34 (2022): 4975–4982, 10.1021/acs.chemmater.2c00224.

[20] H. Ahmed , H. Alijani , A. El‐Ghazaly , et al., “Recovery of Oxidized Two‐Dimensional MXenes Through High Frequency Nanoscale Electromechanical Vibration,” Nature Communications 14 (2023): 3, 10.1038/s41467-022-34699-3. PMC9810719 36596770

[21] P. H. Nguyen , D. H. Nguyen , D. Kim , et al., “Regenerating MXene by a Facile Chemical Treatment Method,” ACS Applied Materials & Interfaces 14 (2022): 51487–51495, 10.1021/acsami.2c13993. 36326902

[22] M. Dadashi Firouzjaei , S. K. Nemani , M. Sadrzadeh , E. K. Wujcik , M. Elliott , and B. Anasori , “Life‐Cycle Assessment of Ti_3_C_2_T_x_ MXene Synthesis,” Advanced Materials 35 (2023): 2300422, 10.1002/adma.202300422. 37095074

[23] K. Maleski , C. E. Shuck , A. T. Fafarman , and Y. Gogotsi , “The Broad Chromatic Range of Two‐Dimensional Transition Metal Carbides,” Advanced Optical Materials 9 (2021): 2001563, 10.1002/adom.202001563.

[24] C. Shang , H. Nan , C. Bai , et al., “The PEEK Sizing Agents Modified by Different Dimensions Nanomaterials of MXene and CNTs for Enhancing the Interfacial and Mechanical Properties of Carbon fiber Composites,” Colloids Surf A: Physicochem Eng Aspects 726 (2025): 137997.

[25] L. Yin , Y. Yang , H. Yang , et al., “Rapid Foaming of Dense MXene Films Induced by Acid‐base Neutralization Reaction,” Cell Reports Physical Science 4 (2023): 101421, 10.1016/j.xcrp.2023.101421.

[26] F. Xia , J. Lao , R. Yu , et al., “Ambient Oxidation of Ti_3_C_2_ MXene Initialized by Atomic Defects,” Nanoscale 11 (2019): 23330–23337, 10.1039/C9NR07236E. 31793604

[27] M. J. Li , Z. Y. Chi , Y. C. Wu , and R. Riman , “Morphology, Chemical Composition and Phase Transformation of Hydrothermal Derived Sodium Titanate,” Journal of the American Ceramic Society 95 (2012): 3297–3304, 10.1111/j.1551-2916.2012.05330.x.

[28] A. Nakahira , T. Kubo , and C. Numako , “Formation Mechanism of TiO_2_‐derived Titanate Nanotubes Prepared by the Hydrothermal Process,” Inorganic Chemistry 49 (2010): 5845–5852, 10.1021/ic9025816. 20527822

[29] F. Shahzad , M. Alhabeb , C. B. Hatter , et al., “Electromagnetic Interference Shielding With 2D Transition Metal Carbides (MXenes),” Science 353 (2016): 1137–1140, 10.1126/science.aag2421. 27609888

[30] J. Xu , Z. Zhou , T. Yang , et al., “MXene‐Based Oxygen Electrocatalysts: Mechanistic Insights, Property Tuning Strategies, and Prospects Toward Practical Applications,” Advanced Materials 37 (2025): 12724, 10.1002/adma.202512724. 40874452

[31] J. Zhao , C. Tu , W. Sun , et al., “The Catalytic Combustion of CH_2_ Cl_2_ Over SO_4_ ^2−^–Ti_x_ Sn_1−x_ Modified with Ru,” Catalysis Science & Technology 10 (2020): 742–756, 10.1039/C9CY01831J.

[32] J. Zhang , X. Zhang , W. Sun , W. Zhou , and W. Yue , “Chemical Scissor‐Enabled Synthesis of Ti_3_C_2_T* _x_ * MXene Nanowires for Selective Oxygen Reduction to Hydrogen Peroxide,” Applied Surface Science 648 (2024): 159068, 10.1016/j.apsusc.2023.159068.

[33] J. Xu , R. S. Longchamps , X. Wang , et al., “Nucleophilic Substitution Enables MXene Maximum Capacitance and Improved Stability,” Advanced Functional Materials 34 (2024): 2408892, 10.1002/adfm.202408892.

[34] A. Sarycheva and Y. Gogotsi , “Raman Spectroscopy Analysis of the Structure and Surface Chemistry of Ti_3_C_2_T_x_ MXene,” Chemistry of Materials 32 (2020): 3480–3488, 10.1021/acs.chemmater.0c00359.

[35] G. Wang , A. Chen , Y. Chen , et al., “Advancements in Electrochemical Synthesis: Expanding from Water Electrolysis to Dual‐Value‐Added Products,” eScience 5 (2025): 100333.

[36] X. Zhao , A. Vashisth , J. W. Blivin , et al., “pH, Nanosheet Concentration, and Antioxidant Affect the Oxidation of Ti_3_C_2_T_x_ and Ti_2_CT_x_ MXene Dispersions,” Advanced Materials Interfaces 7 (2020): 2000845, 10.1002/admi.202000845.

[37] A. Coste , A. Poulesquen , O. Diat , J. F. Dufreche , and M. Duvail , “Investigation of the Structure of Concentrated NaOH Aqueous Solutions by Combining Molecular Dynamics and Wide‐angle X‐ray Scattering,” Journal of Physics and Chemistry B 123 (2019): 5121–5130. 10.1021/acs.jpcb.9b00495 31141363

[38] M. Hellström and J. Behler , “Structure of Aqueous NaOH Solutions: Insights from Neural‐Network‐based Molecular Dynamics Simulations,” Physical Chemistry Chemical Physics 19 (2017): 82–96, 10.1039/C6CP06547C. 27805193

[39] Z. Fan , H. He , J. Yu , et al., “Binder‐Free Ti_3_C_2_T* _x_ * MXene Doughs With High Redispersibility,” ACS Materials Letters 2 (2020): 1598–1605, 10.1021/acsmaterialslett.0c00422.

[40] L. Yin , Y. Wang , Z. Zhao , et al., “Rapid and Reversible Semi‐solidification of MXene Nanosheets via Efficient Capture for Industrial Applications,” Carbon 234 (2025): 119966, 10.1016/j.carbon.2024.119966.

[41] X. Zhang , X. Liu , Q. Liu , et al., “Reversible Constrained Dissociation and Reassembly of MXene Films,” Advanced Science 11 (2024): 2309171. 38582527 10.1002/advs.202309171 PMC11186054

[42] J. Tang , T. Mathis , X. Zhong , et al., “Optimizing Ion Pathway in Titanium Carbide MXene for Practical High‐Rate Supercapacitor,” Advanced Energy Materials 11 (2021): 2003025, 10.1002/aenm.202003025.

[43] J. Tang , T. S. Mathis , N. Kurra , et al., “Tuning the Electrochemical Performance of Titanium Carbide MXene by Controllable In Situ Anodic Oxidation Controllable in Situ Anodic Oxidation,” Angewandte Chemie International Edition 58 (2019): 17849–17855, 10.1002/anie.201911604. 31574196

[44] X. Huang , J. Huang , D. Yang , and P. Wu , “A Multi‐Scale Structural Engineering Strategy for High‐Performance MXene Hydrogel Supercapacitor Electrode,” Advanced Science 8 (2021): 2101664, 10.1002/advs.202101664. 34338445 PMC8456213

[45] K. Li , J. Zhao , A. Zhussupbekova , et al., “4D Printing of MXene Hydrogels for High‐Efficiency Pseudocapacitive Energy Storage,” Nature Communications 13 (2022): 6884, 10.1038/s41467-022-34583-0. PMC9653467 36371429

[46] J. Li , X. Yuan , C. Lin , et al., “Achieving High Pseudocapacitance of 2D Titanium Carbide (MXene) by Cation Intercalation and Surface Modification,” Advanced Energy Materials 7 (2017): 1602725, 10.1002/aenm.201602725.

[47] M. Sadiq , Q. N. K. Hoang , A. Kishlock , et al., “MXene‐montmorillonite Nanocomposites‐based Scaffold Sensors for Early Pancreatic Cancer Diagnosis,” Cancer Plus 6 (2024): 3793.

[48] Y. Tan , M. Yi , Z. Zhu , et al., “Carbon‐coated MoSe_2_/Mo_2_CT* _x_ * (MXene) Heterostructure for Efficient Hydrogen Evolution,” Materials Science and Engineering: B 271 (2021): 115239, 10.1016/j.mseb.2021.115239.

[49] Y. Xia , T. S. Mathis , M.‐Q. Zhao , et al., “Thickness‐independent Capacitance of Vertically Aligned Liquid‐crystalline MXenes,” Nature 557 (2018): 409–412, 10.1038/s41586-018-0109-z. 29769673

[50] M. R. Lukatskaya , S. Kota , Z. Lin , et al., “Ultra‐high‐rate Pseudocapacitive Energy Storage in Two‐dimensional Transition Metal Carbides,” Nature Energy 2 (2017): 17105, 10.1038/nenergy.2017.105.

[51] Z. Fang , C. Peng , Q. Zhou , and Z. Liu , “Electrocatalytic Hydrogen Peroxide Production: Advances, Challenges, and Future Perspectives,” Chemical Record 25 (2025): 202500066. 10.1002/tcr.202500066 40904237

[52] P. Zhang , J. Li , D. Yang , R. A. Soomro , and B. Xu , “Flexible Carbon Dots‐Intercalated MXene Film Electrode with Outstanding Volumetric Performance for Supercapacitors,” Advanced Functional Materials 33 (2022): 2209918, 10.1002/adfm.202209918.

[53] Q. Liang , K. Liu , T. Xu , et al., “Interfacial Modulation of Ti_3_C_2_T* _x_ * MXene by Cellulose Nanofibrils to Construct Hybrid Fibers With High Volumetric Specific Capacitance,” Small 20 (2024): 2307344, 10.1002/smll.202307344. 38133516

[54] H. Wang , Y. Wang , J. Chang , et al., “Nacre‐Inspired Strong MXene/Cellulose Fiber with Superior Supercapacitive Performance via Synergizing the Interfacial Bonding and Interlayer Spacing,” Nano Letters 23 (2023): 5663–5672, 10.1021/acs.nanolett.3c01307. 37310991

[55] M. Han , Y. Liu , R. Rakhmanov , et al., “Solution‐Processed Ti_3_C_2_T_x_ MXene Antennas for Radio‐Frequency Communication,” Advanced Materials 33 (2021): 2003225, 10.1002/adma.202003225. PMC9119193 33251683

[56] Y. Guan , L. Yang , C. Chen , R. Wan , C. Guo , and P. Wang , “Regulable Crack Patterns for the Fabrication of High‐performance Transparent EMI Shielding Windows,” iScience 28 (2025): 111543. 39807168 10.1016/j.isci.2024.111543 PMC11729037

[57] H. Xu , Y. Wang , M. Liu , and Y. Zhai , “Alternating Multilayered Ti_3_C_2_T* _x_ */Co Sandwich with Co Frosting for Superior Electromagnetic Wave Absorption Performance and Infrared Stealth Ability,” ACS Applied Materials and Interfaces 17 (2025): 47679–47695. 40771006 10.1021/acsami.5c09658

[58] A. Iqbal , F. Shahzad , K. Hantanasirisakul , et al., “Anomalous Absorption of Electromagnetic Waves by 2D Transition Metal Carbonitride Ti_3_CNT* _x_ * (MXene),” Science 369 (2020): 446–450, 10.1126/science.aba7977. 32703878

[59] X. Wang , X. Hu , Z. Liu , et al., “Interpenetrating Double‐network ANF/MXene‐K^+^ Aerogels Enable Integrated Electromagnetic Interference Shielding, Infrared Camouflage, and Joule Heating in Adaptive Multifunctional Systems,” Nano Research 18 (2025): 94907702.

[60] J. Liang , F. Ye , Q. Song , et al., “Genetic Algorithm Designed Multilayered Si_3_N_4_ Nanowire Membranes Hybridized by Dielectric Wide‐range Tunable CVD Graphene Skin for Broadband Microwave Absorption,” Composites Part B: Engineering 297 (2025): 112298.

[61] J. Liang , F. Ye , Q. Song , et al., “In‐Plane Heterogeneous Structure‐Boosted Interfacial Polarization in Graphene for Wide‐band and Wide‐temperature Microwave Absorption,” Chemical Engineering Journal 497 (2024): 154307.

[62] S. Wan , X. Li , Y. Chen , et al., “High‐strength Scalable MXene Films Through Bridging‐induced Densification,” Science 374 (2021): 96–99, 10.1126/science.abg2026. 34591632

[63] M. Han , D. Zhang , A. Singh , et al., “Versatility of Infrared Properties of MXenes,” Materials Today 64 (2023): 31–39.

[64] Y. Li , C. Xiong , H. Huang , et al., “2D Ti_3_C_2_T* _x_ * MXenes: Visible Black but Infrared White Materials,” Advanced Materials 33 (2021): 2103054, 10.1002/adma.202103054. 34463370

[65] Z. Deng , P. Jiang , Z. Wang , L. Xu , Z.‐Z. Yu , and H.‐B. Zhang , “Scalable Production of Catecholamine‐Densified MXene Coatings for Electromagnetic Shielding and Infrared Stealth,” Small 19 (2023): 2304278, 10.1002/smll.202304278. 37431209

[66] K. Li , C. Lin , G. Liu , et al., “Stepless IR Chromism in Ti_3_C_2_T* _x_ * MXene Tuned by Interlayer Water Molecules,” Advanced Materials 36 (2024): 2308189, 10.1002/adma.202308189. 38014765

[67] M. Downes , C. E. Shuck , B. McBride , J. Busa , and Y. Gogotsi , “Comprehensive Synthesis of Ti_3_C_2_T* _x_ * From MAX Phase to MXene,” Nature Protocols 19 (2024): 1807–1834, 10.1038/s41596-024-00969-1. 38504139

[68] K. R. G. Lim , M. Shekhirev , B. C. Wyatt , B. Anasori , Y. Gogotsi , and Z. W. Seh , “Fundamentals of MXene Synthesis,” Nature Synthesis 1 (2022): 601–614, 10.1038/s44160-022-00104-6.

[69] A. Lipatov , M. Alhabeb , M. R. Lukatskaya , A. Boson , Y. Gogotsi , and A. Sinitskii , “Effect of Synthesis on Quality, Electronic Properties and Environmental Stability of Individual Monolayer Ti_3_C_2_ MXene Flakes,” Advanced Electronic Materials 2 (2016): 1600255, 10.1002/aelm.201600255.

[70] G. Kresse and J. Furthmüller , “Efficient Iterative Schemes for Ab Initio Total‐energy Calculations Using a Plane‐wave Basis Set,” Physical Review B 54 (1996): 11169–11186, 10.1103/PhysRevB.54.11169. 9984901

[71] P. E. Blöchl , “Projector Augmented‐wave Method,” Physical Review B 50 (1994): 17953–17979. 10.1103/physrevb.50.17953 9976227

[72] J. P. Perdew , K. Burke , and M. Ernzerhof , “Generalized Gradient Approximation Made Simple,” Physical Review Letters 77 (1996): 3865–3868, 10.1103/PhysRevLett.77.3865. 10062328

[73] H. Sun , “COMPASS: An ab Initio Force‐Field Optimized for Condensed‐Phase Application Overview With Details on Alkane and Benzene Compounds,” The Journal of Physical Chemistry B 102 (1998): 7338–7364, 10.1021/jp980939v.

