# Supplementary material for: Ion‐Confinement‐Assisted Erasure Purifies Oxidized MXene for Reuse
Source: Adv Sci (Weinh). 2026 Jan 25;13(18):e22266. doi: 10.1002/advs.202522266 (PMC13042617; doi:10.1002/advs.202522266)
Supplement: Supplementary file 1 — Supporting File: advs73995‐sup‐0001‐SuppMat.docx. [file ADVS-13-e22266-s001.docx]

Supporting Information

Ion-Confinement-Assisted Erasure Purifies Oxidized MXene for Reuse

*Xuefeng Zhang, Yujia Wu, Huayu Xu, Jinyi Ye, Yanfang Yang, Haohan Wu, Jiayi Lu, Hao Li, Dongxiao Kan, Jingfeng Wang, Hui Kang, and Zhimin Fan*

**Supporting Figures**

**Figure S1.** (a) Schematic illustration of the high-quality monolayer Ti_3_C_2_T*_x_* MXene prepared by a modified minimally intensive layer delamination method. Optical appearance of (b) MXene Ti_3_C_2_T*_x_* dispersion and (c) its Tyndall effect after dilution.

Figure S2. SEM images of Ti_3_AlC_2_ MAX phase.

Figure S3. TEM images and elemental mapping of Ti_3_C_2_T*_x_* MXene nanosheets.

Figure S4. (a) Schematic illustration and macroscopic view of vacuum-filtered Ti_3_C_2_T*_x_* MXene film. (b) Surface and (c) cross-sectional morphology of the MXene film.

**Figure S5.** Raman spectrum of the Ti_3_C_2_T*_x_* MXene.

Figure S6. Zeta potential and optical appearance of MXene after 2 months of storage and subsequent erasure-purification. The sample stored for 2 months is denoted as MXene-2S, the sample after alkaline hydrothermal treatment as MXene-2L, and the final purified sample as MXene-2R.

Figure S7. Size distributions of TiO_2_ in (a) MXene-1S and (b) MXene-2S.

Figure S8. XRD patterns of MXene-2S, MXene-2L, and MXene-2R.

Figure S9. TEM images and elemental mapping of (a) MXene-2S and (b) MXene-2L.

Figure S10. Comparison of Na contents in MXene-1L, MXene-1R, MXene-2L, and MXene-2R.

Figure S11. TEM images and elemental mapping of MXene-2R.

Figure S12. (a) FTIR spectra of MXene, MXene-1S, MXene-1L, and MXene-1R. (b) FTIR spectra of MXene-2S, MXene-2L, and MXene-2R.

**Figure S13.** UV-vis absorbance spectra of (a) MXene, MXene-1S, MXene-1L and MXene-1R; (b) MXene-2S, MXene-2L, and MXene-2R.

Figure S14. (a) Raman spectra and (b) Ti 2p binding energy spectra of MXene-2S, MXene-2L, and MXene-2R.

Figure S15. (a) Surface and (b) cross-section of MXene-1S. (c) Surface and (d) cross-section of MXene-1L. (e) Surface and (f) cross-section of MXene-1R.

Figure S16. Densities of films assembled from MXene, MXene-1S, MXene-1L, MXene-1R, and MXene-2R.

Figure S17. (a) Optical image and (b) bent morphology of MXene-1R film.

Figure S18. SEM images of (a) MXene-2S and (b) MXene-2L. (c) Surface and (d) cross-sectional SEM images of MXene-2R.

Figure S19. Morphologies and particle size distributions of nanosheets for (a) MXene, (b) MXene-1R, and (c) MXene-2R.

**Figure S20.** (a) Zeta potentials of MXene, MXene-1R and MXene-2R. (b) Schematic illustration of hydrated sodium ions at the MXene surface in sodium hydroxide solutions with different concentrations.

Figure S21. (a) Photograph of a large-area film assembled from MXene-1R. (b) Electrical conductivities of MXene-1R, MXene-1S, MXene-1L, MXene, and MXene-2R.

Figure S22. Comparison of the process flow of the erasure-recovery strategy proposed in this work with previously reported approaches that show potential for scalable MXene production.^[1-6]^

**Figure S23.** (a) Schematic illustration of recovery TiO_2_ quantum dot. SEM images of (b) TiO_2_-1R and (c)TiO_2_-2R. (d) O/Ti atomic ratios and elemental compositions of TiO_2_-1R and (c) TiO_2_-2R. (e) Optical image of TiO_2_-2R.

**Figure S24.** TEM images and element mappings of (a) TiO_2_-1R and (b) TiO_2_-2R. (c) XRD patterns and (d) Raman spectra of TiO_2_-1R and TiO_2_-2R.

Figure S25. SEM images of MXene films assembled after erasure and purification under different NaOH concentrations: (a) 10 M (10M-NaOH), (b) 5 M (5M-NaOH), and (c) 2 M (2M-NaOH).

Figure S26. (a) XRD patterns and (b) high-resolution Ti 2p spectra of MXene samples purified under different NaOH concentrations.

Figure S27. (a) XRD patterns and (b) high-resolution Ti 2p spectra of MXene samples purified using hydrochloric acid at different concentrations: 10 M (10M-HCl), 5 M (5M-HCl), 2 M (2M-HCl) and 0.5 M (05M-HCl).

**Figure S28.** SEM images of MXene films purified with hydrochloric acid at different concentrations: (a) 10 M, (b) 5 M, (c) 2 M, and (d) 0.5 M.

Figure S29. SEM images of accordion-like multilayer MXene particles: (a) prepared by HF etching (MXene-P), (b) oxidized after aging (MXene-P-S), and (c) purified after erasure treatment (MXene-P-R).

Figure S30. (a) XRD patterns and (b) high-resolution Ti 2p spectra of MXene-P and oxidized MXene-P-S.

Figure S31. Surface SEM images of (a) the failed MXene film (MXene-Film-S) and (b) the restored MXene film after erasure and purification (MXene-Film-R).

Figure S32. Surface SEM images of (a) the failed GO/MXene composite film (MXene-G-S) and (b) the GO/MXene composite film after erasure and purification (MXene-G-R).

Figure S33. Surface SEM images of (a) the failed MXene film after exposure to sulfuric acid electrolyte (MXene-H-S) and (b) the corresponding MXene film after erasure and purification (MXene-H-R).

Figure S34. (a) XRD patterns and (b) high-resolution Ti 2p XPS spectra of MXene-Film-S, MXene-G-S, and MXene-H-S.

Figure S35. Cyclic voltammetry (CV) curves of (a) MXene, (b) MXene-1S, (c) MXene-1L, and (d) MXene-1R at various scan rates.

Figure S36. GCD curves of (a) MXene, (b) MXene-1S, (c) MXene-1L, and (d) MXene-1R at different current densities.

Figure S37. (a) CV curves of MXene-2R at different scan rates; (b) galvanostatic charge–discharge (GCD) curves of MXene-2R at different current densities; (c) specific capacitance of MXene-2R as a function of scan rate; (d) specific capacitance of MXene-2R as a function of current density.

**Figure S38.** Enlarged view of the (002) diffraction peak of the uncleaned MXene-1R film after cycling.

Figure S39. CV curves of symmetric devices assembled from (a) MXene, (b) MXene-1S, (c) MXene-1L, and (d) MXene-1R.

Figure S40. GCD curves of symmetric devices assembled from (a) MXene, (b) MXene-1S, (c) MXene-1L, and (d) MXene-1R.

**Figure S41.** Ragone plots of MXene after erasure compared with several reported supercapacitors.^[7-11]^

Figure S42. Planar view of the EM RL of MXene.

Figure S43. Infrared stealth performance of MXene-1R film continuously placed on a heating stage at (a) 40°C, (b) 120°C, and (c) 240°C.

**Table S1.** The electrochemical performance of erasure-recovered MXene compared with other previously reported MXene-based electrodes.

| **Electrodes** | **Current density**  **(A g^-1^)** | **Volume specific capacitance**  **(F cm^-3^)** | **scan rate**  **(mV s^-1^)** | **Volume specific capacitance**  **(F cm^-3^)** | **electrolyte** | **Reference** |
| --- | --- | --- | --- | --- | --- | --- |
| Carbon Dots-Intercalated MXene Film | 1 | 1244.6 | 5 | 1188.5 | 3M H_2_SO_4_ | 12 |
| Liquid-mediated holey MXene film | - | - | 10 | 1274 | 3M H_2_SO_4_ | 13 |
| Black phosphorus/MXene Composite Film | - | - | 2 | 640 | 1MNa_2_SO_4_ | 14 |
| MXene/PANI Composite Film | 1 | 1353 | 2 | 1682 | 3M H_2_SO_4_ | 15 |
| MXene film oxidized by sulfuric acid | - | - | 5 | 1200 | 3 M H_2_SO_4_ | 16 |
| Nanoscale Architecture MXenne | - | - | 2 | 770 | 1M H_2_SO_4_ | 17 |
| Ultradense  MXene Fiber | - | - | 2 | 1661 | 1M H_2_SO_4_ | 18 |
| MXene/CNT@Ni films | 1 | 990.8 | - | -- | 1M H_2_SO_4_ | 19 |
| MXene/MnO_2_ Film | - | - | 5 | 312 | 1M Na_2_SO_4_ | 20 |
| Fe_2_O_3_/MXene aerogel | 1 | 150 | - | - | 3M H_2_SO_4_ | 21 |
| MXene Nanoyarns | - | - | 5 | 440 | 5.5MLiClO_4_ | 22 |
| Nanofluidic MXene Fibers | - | - | 5 | 1360 | 1M MgSO_4_ | 23 |
| **MXene** | **1** | **1103** | **5** | **998** | **3M H_2_SO_4_** | **This work** |
| **MXene-1S** | **1** | **459** | **5** | **465** | **3M H_2_SO_4_** | **This work** |
| **MXene-1L** | **1** | **897** | **5** | **853** | **3M H_2_SO_4_** | **This work** |
| **MXene-1R** | **1** | **1801** | **5** | **1725** | **3M H_2_SO_4_** | **This work** |
| **MXene-2R** | **1** | **1604** | **5** | **1550** | **3M H_2_SO_4_** | **This work** |

[1] Y. Wang, B. Zhou, Q. Tang, Y. Yang, B. Pu, J. Bai, J. Xu, Q. Feng, Y. Liu, W.Yang, *Adv. Mater.* **2024**, *36*, 2410736.
[2] N. Chen, Z. Duan, W. Cai, Y. Wang, B. Pu. H. Huang, Y. Xie, Q. Tang, H. Zhang, W. Yang, *Nano Energy* **2023**, *107,* 108147.
[3] C. E. Shuck, Y. Gogotsi, Chem. Eng. J. **2020**, *401*,125786.
[4] Z. Huang, J. Qin, Y. Zhu, K. He, H. Chen, H. Y. Hoh, M. Batmunkh, T. M. Benedetti, Q. Zhang, C. Su, S. Zhang, Y. L. Zhong, *Carbon Energy* **2023**, *5*, 295.
[5] C. E. Shuck, M. Han, K. Maleski, K. Hantanasirisakul, S. J. Kim, J. Choi, W. E. B. Reil, Y. Gogotsi, al. ACS Appl. Nano Mater. **2019**, *2,* 3368-3376.
[6] M. D. Firouzjaei, S. K. Nemani, M. Sadrzadeh. E. K. Wujcik, M. Elliott, B. Anasori, *Adv. Mater.* **2023**, 35, 2300422.
[7] J. Ma, S. Zheng, Y. Cao, Y. Zhu, P. Das, H. Wang, Y. Liu, J. Wang, L.Chi, S. Liu, ZS.Wu, *Adv. Energy Mater.* **2021**, 11, 2100746.
[8] W. Cheng, J. Fu, H. Hu, D. Ho, Adv. Sci. **2021**, 8, 2100775.
[9] H. Li, Y. Hou, F. Wang, M.R. Lohe, X. Zhuang, X., L. Niu, X. Feng, *Adv. Energy Mater.* **2016**, 7,1601847.
[10] Q. Liang, K. Liu, T. Xu, Y. Wang, M. Zhang, Q. Zhao, W. Zhong, X. M. Cai, Z. Zhao, C. Si, *Small*, **2024**, 20, 2307344.
[11] P. Zhang, J. Li, D. Yang, R. A. Soomro, B. Xu, *Adv. Funct. Mater.* **2023**, 33, 2209918.
[12] P. Zhang, J. Li, D. Yang, R. A. Soomro, B. Xu, *Adv. Funct. Mater.* **2022**, *33*, 2209918.
[13] Z. Fan, Y. Yang, H. Ma, Y. Wang, Z. Xie, Y. Liu, *Carbon* **2022**, *186*, 150.
[14] J. Yang, Z. Pan, J. Zhong, S. Li, J. Wang, P.Y. Chen, *Energy Storage Mater.* 2021, **36**, 257.
[15] A. VahidMohammadi, J. Moncada, H. Chen, E. Kayali, J. Orangi, C.A. Carrero, M. Beidaghi, *J. Mater. Chem. A* 2018, 6, 22123.
[16] J. Tang, T. Mathis, X. Zhong, X. Xiao, H. Wang, M. Anayee, F. Pan, B. Xu, Y. Gogotsi, *Adv. Energy Mater.* **2021**, *11*, 2003025.
[17] H. Chen, H. Wang, C. Li, *Adv. Mater.* **2022**, *34*, 2205723.
[18] G. He, Z. Cai, S.Xiang, D. Cai, *ACS Applied Nano Mater.* **2021**, *21*, 303.
[19] S. Li, Q. Zhang, L. Liu, J. Wang, L. Zhang, M. Shi, X. Chen, *J. Alloy. Compd.* **2023**, *941*, 168963.
[20] D. Wen, G. Ying, L. Liu, C. Sun, Y. Li, Y. Zhao, Z. Ji, Y. Wu, J. Zhang, J. Zhang, X. Wang, *Adv. Mater. Interfaces* **2021**, *8*, 2101453.
[21] Y. Luo, Y. Tang, X. Bin, C. Xia, W. Que, *Small* **2022**, *18*, 2204917.
[22] A. Levitt, S. Seyedin, J. Zhang, X. Wang, J.M. Razal, G. Dion, Y. Gogotsi, *Small* **2020**, *16*, 2002158.
[23] S. Li, Z. Fan, G. Wu, Y. Shao, Z. Xia, C. Wei, F. Shen, X. Tong, J. Yu, K. Chen, M. Wang, Y. Zhao, Z. Luo, M. Jian, J. Sun, R.B. Kaner, Y. Shao, *ACS nano* **2021**, *15*, 7821.
